# Evaluation of 5 Novel protein biomarkers for the rapid diagnosis of pulmonary and extra-pulmonary tuberculosis: preliminary results

**DOI:** 10.1038/srep44121

**Published:** 2017-03-24

**Authors:** Amit Singh, Anil Kumar Gupta, Krishnamoorthy Gopinath, Pawan Sharma, Sarman Singh

**Affiliations:** 1 Division of Clinical Microbiology & Molecular Medicine, All India Institute of Medical Sciences, New Delhi, India; 2 International Centre for Genetic Engineering and Biotechnology, New Delhi, India

## Abstract

Improved methods are required for the early and accurate diagnosis of tuberculosis, especially in the patients with smear-negative disease. Several biomarkers have been tried but most have shown poor sensitivity or specificity. In present study we aimed to evaluate the diagnostic utility of five novel antigens identified earlier by us. This is an initial study conducted on 250 subjects. The five recombinant antigens, named as rSS1 (Rv2145c), rSS2 (Rv0164), rSS3 (Rv1437), rSS4 (Rv1827) and rSS5 (Rv2970c), were expressed in pQE-30 expression vector, purified and their sero-diagnostic efficacy was evaluated in an unblinded manner using dot-blot and ELISA methods. The sensitivity and specificity of these novel antigens were compared with commercially available standard esat6 and 38 kDa antigens. Bacteriologically confirmed TB patients, non-TB disease controls and healthy individuals were included. which are based on novel antigen or novel technology, Area under curve (AUC) of the selected antigens were 0.98 (0.98–0.99) for rSS1, 0.88 (0.84–0.92) for rSS2, 0.88 (0.84–0.92) for rSS3, 0.95 (0.93–0.98) for rSS4 and 0.99 (0.98–1.0) for rSS5. Receiver operative characteristic (ROC) curve showed highly significant difference between TB and healthy subjects (p = <0.001). These initial findings, show that the recombinant antigens rSS1, rSS4 and rSS5 could be used as highly potential biomarkers for the serological diagnosis of active TB.

Tuberculosis (TB) still remains as a major health problem in the developing countries and is rated as the number one killer infectious disease. World Health Organization (WHO) estimated 9.6 million new cases and more than 1.5 million deaths annually worldwide and India has the world’s largest tuberculosis epidemics^1^. The biggest hurdle in the control and timely management of tuberculosis is non-availability of rapid, accurate and cost-effective test. In remote areas, TB diagnostics are dependent on the microscopic observation of acid-fast bacilli (AFB) in the clinical samples or by bacteriological culture analysis. Although, AFB-smear staining allows rapid and cost-effective diagnosis of tuberculosis but it has comparatively low sensitivity, especially in children and immuno-compromised patients. The current nucleic acid amplification based tests (NAAT) such as Polymerase Chain Reaction (PCR); Xpert MTB/Rif assay; and Line Probe Assay (LPA) are rapid and sensitive but are expensive. A recently held international TB consortium meeting at New Delhi, outlined the urgent need for newer and better point-of care tests.

The serological tests have been an attractive diagnostic tool due to their convenience, rapidity and easy implementation in the national programmes. These tests have contributed significant role in the early diagnosis and management of several infectious diseases including Human Immunodeficiency Virus (HIV), hepatitis A, B, C, E, Leishmaniasis, malaria etc. However, previous attempts to diagnose TB by serology have met with limited success due to low sensitivity and specificity^2^,^3^. Hence, WHO banned these serological tests in 2011 and later on in 2012 Government of India also banned import manufacturing and sale of these kits^3^,^4^. Therefore rapid, inexpensive and accurate antibody based tests for TB diagnosis are urgently required^5^. Earlier, we had reported few differentially expressed proteins that were over expressed during the active disease and development of drug resistance *in-vivo*^6^. Through bioinformatics analysis, we selected five potential proteins based on their antigenicity. In the present study, we further evaluated these antigens for their diagnostic potential by dot-blot and enzyme linked immuno-sorbent assay (ELISA) in various patient and control groups.

## Results

### Cloning, expression and purification of mycobacterial antigens

**Figure 1:**
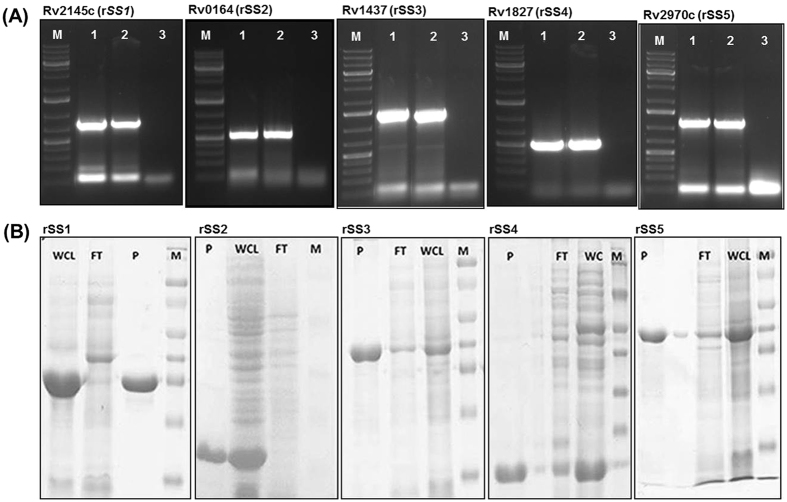
Gene amplification, cloning, expression and purification. (**A**) Agarose gel electrophoresis for PCR products. Lane M, 1 kb DNA Molecular-size marker; lane 1, 2: amplification of respective genes -rSS1 (783 bp), rSS2 (468 bp), rSS3 (489 bp), rSS4 (1239 bp), and rSS5 (1131 bp) from *M. tuberculosis* clinical isolates. lane 3 is negative control. (**B**) SDS-PAGE analyses of purified recombinant proteins (rSS1, rSS2, SS3, rSS4 and rSS5). Proteins were visualized with Coomassie brilliant blue staining. Abbreviations: WCL: Whole Cell Lysate, FT: Flow Through, P: Purified Protein, M: Marker (Protein).

The five novel proteins as described in the material and method section have been named as rSS1 (Rv2145c), rSS2 (Rv0164), rSS3 (Rv1437), rSS4 (Rv1827) and rSS5 (Rv2970c) and a patent has been filed (1752/DEL/2008). The nucleotide sequences of the amplicons were analysed and submitted to GenBank under accession number KC147003, KC147004, KC147005, KC147006 and KC147008, respectively. To generate recombinant antigens, PCR amplification of target genes from genomic DNA of the *Mtb* clinical isolates^7^, was carried out using gene specific primers (Table 1). All the selected genes were successfully amplified with product size of 783 bp (rSS1), 458 bp (rSS2), 1239 bp (rSS3), 489 bp (rSS4) and 1131 bp (rSS5), respectively with appropriate restriction sites (Fig. 1A). The products were cloned and desired proteins were expressed in expression vector as detailed in method section. All recombinant proteins were purified by Ni^2+^-NTA affinity chromatography under denaturing conditions from inclusion bodies except the rSS4 protein which was purified under native condition. The purity (>96%) of N-terminal His-tagged recombinant proteins was analysed by SDS-PAGE (Fig. 1B). The observed molecular weights of rSS1, rSS2, rSS3, rSS4 and rSS5 proteins were approximately 28 kDa, 18 kDa, 42 kDa, 17 kDa and 42 kDa, respectively. The yield of purified recombinant proteins were 3 g (rSS1), 0.7 g (rSS2), 1 g (rSS3), 0.3 g (rSS4) and 0.28 g (rSS5) per litre of the bacterial culture.

### Detection of antibody response against five antigens by immunoblot assay

The immune reactivity of five individual proteins was optimized and used to check diagnostic potential in serum samples of different patient groups using dot-blot assay. Two standard proteins (esat6 and 38 kDa) were kind gift from Prof. VK Chaudhary. The minimum detection concentrations of the purified antigens were found to be 5 ng (rSS1), 10 ng (rSS2), 12.5 ng (rSS3), 10 ng (rSS4), 10 ng (rSS5), 10 ng (38 kDa Ag) and 10 ng (esat6 Ag) of the antigens at 1:200 serum dilution in dot-blot assay. The clinical and laboratory characteristics of the TB patients and healthy controls (HC) included in the study are summarized in Table 2. A total of 250 subjects were included in this study. Of these, 140 were culture confirmed tuberculosis patients [111 pulmonary tuberculosis (PTB) and 29 extra-pulmonary tuberculosis (EPTB)] and 110 controls [60 non-tuberculosis-diseased controls (DC) and 50 healthy controls (HC)]. Of the 111 PTB patients, 15 were HIV-positive and 96 HIV-negative. Among the EPTB patients, five were HIV positive and 15 HIV-negative. Of the 250 subjects 81 (32.4%) had Bacillus Calmette-Guérin (BCG) vaccination with discernible scar and 169 (67.6%) had no vaccination/no discernible scar. Eighty patients (57.1%) were Mantoux/tuberculin skin test (TST) positive positive, while 170 (42.9%) were Mantoux test negative. The control sera (DC plus HC) were included for the assessment of the specificity of selected five proteins in ELISA and dot-blots assay. Of which, 60 subjects had diseases other than tuberculosis, while 50 were HC (with no known past history of TB and TST negative). Eighteen (30%) DC and 14 (28%) HC gave history of *M. bovis* BCG vaccination and were verified by scar. The sera were tested parallally using each recombinant antigen and the results are shown in Tables (3 and 4).

### Dot-blot screening of recombinant antigens for TB diagnosis using dot-blot assay

**Figure 2:**
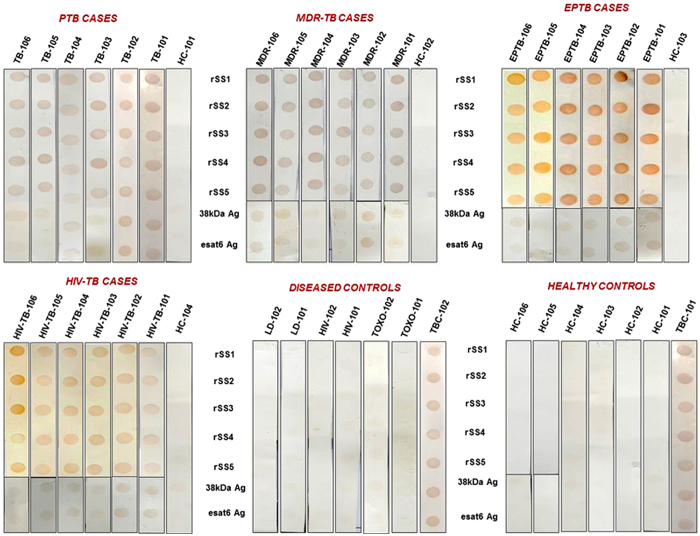
Dot-blot results showing the reactivity of purified recombinant proteins rSS1, rSS2, rSS3, rSS4, rSS5, Ag38 kDa and esat6 with serum samples from patients with extra-pulmonary tuberculosis (EPTB) pulmonary tuberculosis (PTB) HIV-TB, MDR-TB as well as from healthy controls (HC) and diseases controls (DC). Specific binding of the antigen to its antibody is indicated by a dark spot.

The antibody response to the novel antigens was visually analysed (Fig. 2) and results were compared with gold standard MGIT^TM^ 960 culture for sensitivity calculation. The recombinant esat6 and 38 kDa Ag proteins were used as reference antigens. The sensitivity and specificity of esat6 and 38 kDa antigens ranged between 72.4% to 94% and 54.5% to 66.4% respectively. The pooled sensitivity of 38 kDa Ag in PTB, EPTB, MDR-TB cases was 85.6%, 86.2% 94%, while that of esat6 was 85.6%, 72.4%, and 90% respectively. However, in comparison to the reference antigens, the overall performance of our five antigens was much superior. The rSS1, rSS2, rSS3, rSS4, rSS5 antigens showed superior sensitivity and specificity both ranging from 86.2% to 99.1% and 89.1% to 100%, respectively. The detailed sensitivity and specificity values of each antigen are given in Table (3). In PTB cases, the sensitivity of these antigens ranged between 99.1% and 93.7% and specificity in various control groups was between 89.1% and 100%. Interestingly, the rSS1, rSS4 and rSS5 antigens showed 100% sensitivity in HIV-PTB cases (see Supplementary Table 1). In EPTB cases also, the pooled sensitivity and specificity were very high (Table 3). Antigens rSS1, rSS2, rSS4 and rSS5 showed 100% sensitivity in HIV-EPTB cases and smear positive EPTB cases (Supplementary Table 1). In Mantoux positive TB cases, 100% (8/8) sensitivity was observed for rSS5, rSS4 and rSS1 antigens followed by 87.5% for rSS2, rSS3, Ag38 kDa and esat6 antigens (Supplementary Table 2). In smear negative culture positive cases, antigen rSS5 showed maximum sensitivity of 96.0%. The sensitivity and specificity rates of the 5 antigens by dot-blot assay, in MDR-TB cases were 100% while sensitivity of 38 kDa Ag and esat6 Ag were found 94% and 90% respectively (Table 3 and Supplementary Table 3).

### ELISA screening of purified recombinant antigens for TB diagnosis using ELISA test

**Figure 3:**
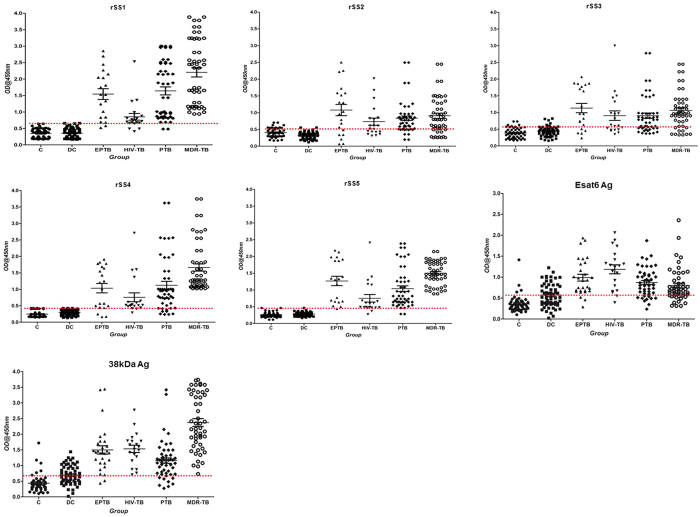
Scatter plots of ELISA results using our novel recombinant antigens and the reference antigens. Recombinant antigens assayed are rSS1, rSS2, rSS3, rSS4, rSS5, Esat-6 and 38 kDa Ag). The serum samples used were from of healthy controls (HC), diseased controls (DC), tuberculosis patient (PTB, EPTB, HIV-TB) and MDR-TB. The scatter plot indicates the antibody level per subject analysed. A dotted horizontal line is included to show the cut-off value for individual antigen.

**Figure 4:**
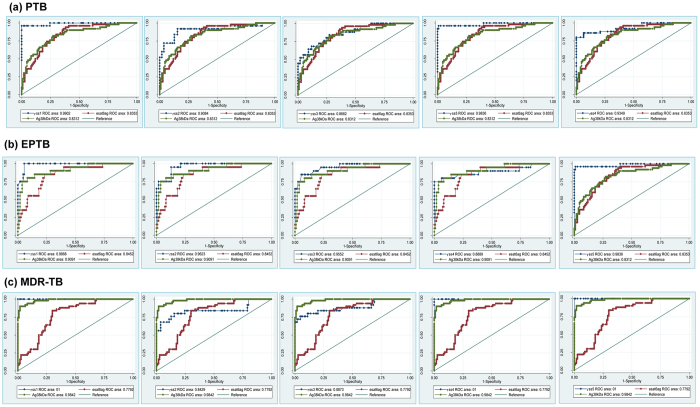
Receiver operative characteristic (ROC) curves of the antibody response against five *M. tuberculosis* recombinant antigens (rSS1, rSS2, rSS3, rSS4, rSS5) with two reference antigens (esat6 and Ag38 kDa) in (**a**) PTB, (**b**) EPTB and (**c**) MDR-TB patients and healthy controls.

The optimum concentration of recombinant antigen yielding high specificity was determined at 25 ng/well for rSS1 and rSS5, 50 ng/well for rSS2, rSS3, rSS4, 38 kDa Ag, esat6 Ag and the serum dilutions of primary and secondary conjugated antibodies were found to be 1:50 and 1:15000, respectively. Using these dilutions all of the 250 serum samples (Table 2) were analysed by indirect ELISA. The cut-off value (Mean ± 2 SD) of the ELISA was determined by area under curve (AUC) from 140 TB patient serum samples and 110 HC and DC serum samples (Fig. 3). The cut-off values were 0.597 for rSS1, 0.489 for rSS2, 0.540 for rSS3, 0.411 for rSS4, 0.410 for rSS5, 0.57 for 38 kDa Ag and 0.56 for esat6 Ag. The AUC were 0.98 (0.98–0.99) for rSS1, 0.88 (0.84–0.92 for rSS2, 0.88 (0.84–0.92) for rSS3, 0.95 (0.93–0.98) for rSS4, 0.99 (0.98–1.0) for rSS5 0.90(0.88–0.92) for 38 kDa Ag and 0.81(0.79–0.83) for esat6 Ag (Fig. 4).

The highest sensitivity and specificity values for these recombinant antigens were 96.5% & 98.2% for rSS5, 96.4% & 97.3% for rSS1, 73% & 87.3% for rSS2, 75.9% & 86.4% for rSS3, 91.9% and 94.5% for rSS4, respectively (Table 4). The overall sensitivity of esat6 & 38 kDa Ag was 81.3% and 91.25%, while specificity was 70.9% and 60.9% respectively. There was a clear difference in the antibody levels observed in healthy controls *vs* TB patients (P_rSS5_ < 0.001, P_rSS1_ < 0.001, P_rSS4_ < 0.001, P_rSS2_ < 0.001 and P_r_SS3 < 0.001, respectively) (Table 4; Fig. 4), However, rSS5, rSS1 and rSS4 antigens showed very good activity over other antigens in MDR-TB cases. The pooled sensitivity rates in PTB cases by ELISA for antigens rSS1, rSS2, rSS3, rSS4 and rSS5 were between 73.0% to 96.4%, (Supplementary Table 4). In EPTB cases, the highest sensitivity (96.5%) and specificity (98.2%) was shown by rSS5, followed by 89.7% & 97.3% for rSS1, 89.7% & 94.5% for rSS4, 89.7% & 70.9% for esat6 Ag, 89.7% & 60.9% for 38 kDa Ag, 75.9% & 86.7% for rSS3, 69.0% & 87.3% for rSS2 (Fig. 4 and Supplementary Table 5). The sensitivity and specificity of five antigens in the MDR-TB patients are given in Supplementary Table (6). In comparison to reference antigens, our three antigens (rSS1, rSS4, rSS5) showed extraordinary performance in PTB cases (Supplementary Table 4). In EPTB cases, only rSS5 antigen showed very high sensitivity (Fig. 4 and Supplementary Table 5).

## Discussion

Rapid and accurate diagnosis of tuberculosis is crucial to facilitate early treatment initiation, reducing disease transmission and preventing emergence of drug resistant strains. The currently used methods are either insensitive, time consuming, costly or require high technical skill and laboratory infrastructure. In search of rapid and cost-effective diagnosis of tuberculosis, serological test have been considered an attractive option. In recent past decades, a variety of serological assays have been developed such as latex agglutination, ELISA, indirect immunofluorescence and rapid immunochromatographic tests^8^,^9^,^10^,^11^. The rapid serological tests have successfully been used to combat several infectious diseases like malaria, leishmaniasis, HIV, hepatitis viral infections etc. to name a few. These serological tests, if developed successfully for tuberculosis, will have an additional advantage over all other currently used tests, specially in patients who are unable to produce sputum (such as children and HIV/AIDS patients) and patients with EPTB. These serological tests will also have potential to develop point of care (POC) tests that can reach to lowest levels of health services.

To develope the serological tests, for the diagnosis of tuberculosis, researchers have used both crude as well recombinant antigens^8^,^9^,^12^. The performance of these tests has been evaluated using single as well as cocktail of multiple antigens^13^,^14^,^15^,^16^. The most commonly used and commercially available antigens have been the esat6 and *Mtb* 38 kDa recombinant antigens. However, this antigen showed sensitivity of only 47% and specificity of 94% in smear positive patients, in studies carried out earlier^13^. In present study also the performance of this antigen was far inferior to our newly described antigens. Another recombinant protein used earlier was malate synthase and MPT51. At laboratory level, the malate synthase showed the specificity of 98% and sensitivity of 73% in sputum smear positive patients^17^,^18^. The MPT51 protein showed poor sensitivity rates of 59% in HIV-negative TB patients and 58% in HIV-TB patients^19^,^20^. Its sensitivity and specificity rates reported by others were between 74 to 80% and 34 to 74% respectively^21^,^22^. Recently, Feng *et al*.^23^ reported that a polyprotein comprising of 38 kDa and MPT64 are suitable for diagnosing active tuberculosis with sensitivity and specificity of 70.4% and 91.5% respectively. A recombinant TbF6 antigen was generated by fusion of four distinct antigens (38 kDa, CFP-10, MTB8, and MTB48) in a single protein. However, its sensitivity in sputum smear positive patients remained below 70% only^17^,^19^,^24^,^25^. These and several other antigens were used to develop commercial ELISA and rapid diagnostic test (RDT) kits, despite giving low sensitivity and specificity^3^. Prompted by concerns raised by academia in 2008, the WHO special programme for Research and Training in Tropical Diseases (TDR) performed an evaluation of 19 commercially available TB diagnostic kits^26^. The meta-analysis of these kit evaluation results showed the sensitivity of 1% to 60% and specificity of 53% to 99% under field conditions. Several other systematic reviews and original evaluation studies also showed similar findings^3^,^4^,^5^,^17^,^27^.

Our antigens provided outstanding sensitivity and specificity, not reported earlier. This could most likely be due to the fact that in all previous studies recombinant antigens were prepared from the laboratory maintained H37Rv strain, while in our study we used a well characterized but fresh clinical isolate which developed *in-vivo* multidrug resistance^6^,^7^. We consider that this was a major turning point in the development of these antigens. It has been documented that expression levels as well as the characters of proteins expressed by the H37Ra, H37Rv, and clinical isolates are significantly different^28^. It is also important to mention that the diagnostic potential of the five recombinant proteins which we used in this study, has never been explored on such a scale for the diagnosis of tuberculosis^29^.

For comparison we included esat6 and 38 kDa antigens as reference proteins. In fact while comparing the sensitivity and specificity of our antigens with these two reference antigens, we found that antigen rSS1, rSS4 and rSS5 are highly significantly superior to the esat6 and 38 kDa Ag or other previously published studies^30^. In comparison to reference antigen (esat6 and 38 kDa Ag), We noticed great difference in the specificity of rSS1, rSS2, rSS3, rSS4, rSS5 antigens, though only three antigens (rSS1, rSS4, rSS5) showed significant difference in the sensitivity. We found that in previous evaluation studies, several kits were marketed without evaluating the specificity of these antigens on disease controls (i.e. HIV, leishmaniasis, toxoplasmosis, cancer, and diabetes) in whom these commercial kits failed badly. However, we have included not only healthy controls but also various disease controls in this study. Most importantly, three recombinant antigens (rSS5, rSS1 and rSS4) showed very high utility in MDR-TB cases. This is an important finding of our study and we propose that these three antigens can be used not only for diagnosis of TB but can help in the ruling out the disease severity. We found higher sensitivity of all these antigen in dot-blot as compared to ELISA. The reason for this could be higher cut-off taken in ELISA while in dot-blot any visible dot was considered as positive. Likelihood ratio (LR) of positive test is also an important statistical method to better evaluate the diagnostic test. In our study, LR of positive test values was very high (ranging from 5.5–53.5). The areas under the ROC curve (AUC) showed an excellent diagnostic efficacy of our recombinant antigens (Table 4). The data also shows that our antigens do not cross react with *M. bovis* (BCG) vaccine. The antigen rSS4 is conserved hypothetical protein which stimulates T-cell response in the host. The rSS5 is a probable lipase/esterase LipN while rSS2 is an essential hypothetical protein^29^. The rSS3 is a phosphoglycerate kinase^6^ involved in the second phase of glycolysis and to best of our knowledge, none of these antigen has been used for serodiagnosis of tuberculosis in India. Even though, the study showed very high utility of three antigen in the detection of active TB, but study had some limitations; all antigens were not tested with other disease control groups *i.e.* house hold contacts, latent TB, other pulmonary diseases such as asthma, bronchitis, pneumonia, allergies and also need to be evaluated in different geographical regions of world before commercialization.

Therefore, we can conclude that these results convince us that at least 3 of these five antigens can successfully be used for screening all the suspected cases of active tuberculosis, though not for confirming the diagnosis. We are in process of developing semi-quantitative rapid diagnostics tests (RDTs) in a way that these biomarkers can be used to detect and predicting tuberculosis, by measuring the high expression of these antigens. We strongly believe that rapid tests developed from these antigens can meet an urgent requirement of triage test for all forms of tuberculosis.

## Material and Methods

### Cloning of the over-expressing genes

To express the desired antigens, the genomic DNA of *Mtb* clinical isolates (AIIMS/LM/SS/TB-1920/06) was used as the template for PCR reactions using gene specific primers (Table 1) as reported earlier^7^. The conditions for PCR were as follows: a 50 μl PCR mixture were comprised of 10 mM Tris-Cl (pH 8.2); 50 mM MgCl_2_, deoxynucleoside triphosphate (200 μM each), 1.0 U of Taq DNA polymerase, 0.5 μl of each primer and 100 ng of genomic DNA. The reaction mixture was subjected to initial denaturation at 94 °C for 10 min followed by 30 amplification cycles of 94 °C for 1 min, annealing (rSS1 at 53 °C; rSS2 at 64 °C; rSS3 at 58 °C; rSS4 at 65 °C and rSS5 at 58 °C) for 45 sec, amplification at 72 °C for 45 sec and final extension at 72 °C for 10 min in a PTC-100 thermal cycler (MJ Research, USA).

Purified PCR product of each gene was cloned in to the pGEMT easy cloning vector and transformed in *E. coli* JM109 strain as per manufacturer instruction and plated out in appropriate antibiotic containing plates. Blue-white selection method was used for clones screening. Plasmids were extracted from randomly selected clones and subjected to restriction digestion by *HindIII* and *BamH*I restriction enzymes for clone confirmation. Gel purified inserts were sub-cloned into pQE-30 expression vector and transformed in *E. coli* M15 strain. All recombinant clones were selected in 100 μg/ml ampicillin and 50 μg/ml kanamycin containing Luria-Bertani (LB) agar plate. Plasmids were again extracted from all selected clones and presence of insert was confirmed by DNA sequencing.

Recombinant clones were cultured overnight at 37 °C in LB broth containing appropriate antibiotics. Overnight grown culture was inoculated into fresh LB medium containing antibiotics and incubated at 37 °C with shaking at 225 rpm until OD (600 nm) reached at 0.6. Thereafter, protein expression was induced by 1 mM isopropyl-β-D-1-thiogalactopyranoside (IPTG) at the mid-exponential phase of growth. Cultures were grown for an additional 4 h with shaking at 37 °C for proteins expression. Bacterial pellets were harvested by centrifugation at 12,000 rpm for 5 min and stored at −80 °C until use. Pellets were re-suspended in lysis buffer (20 mM Tris (pH-8.0), 150 mM NaCl and 1 mM phenylmethylsulfonyl fluoride (PMSF) and disrupted by sonication followed by centrifugation at 12000 rpm for 30 min at 4 °C. Recombinant proteins were purified under native and denaturing conditions by using Ni^2+^ NTA metal-ion-affinity chromatography as per manufacturer’s instructions (Qiagen, Germany). Purity level were analysed through sodium dodecyl sulphate polyacrylamide gel electrophoresis (SDS-PAGE). Purified proteins were dialyzed against 20 mM Tris, pH 8.0, and concentrated using Amicon Ultra 10 kDa/3 kDa – cut off centrifugal filters (Millipore, India). The concentration of purified proteins was quantified by Bradford protein assay (Bio-Rad, USA).

### Recombinant esat6 Ag and 38 kDa Ag proteins

The recombinant esat6 Ag and 38 kDa Ag were kind gifts from CIIDRET, University of Delhi South Campus, New Delhi. The preparation of esat6 is described previously^31^,^32^. The 38 kDa antigen was produced as N-terminal deca-histidine-tagged protein using T7 promoter-based expression system as described previously for hexa-histidine-tagged 38 kDa^33^.

### Study population and serum sample collection

The study was conducted from 2009–2015. Institutional ethics committee of the All India Institute of Medical Sciences (AIIMS), New Delhi approved the study (Ref No. T-9/31.7.2009). The participants were included after informed and written consent to participate in this study. A total of 250 subjects [TB patients (n = 140), DC (n = 60) and HC (n = 50)] were eligible for the evaluation of the diagnostic performance of five recombinant antigens. Of 140 culture confirmed TB cases, 111 were PTB PTB cases and 29 samples were from EPTB cases included 50 MDR-TB drug susceptibility (DST) confirmed cases (n = 46 PTB, n = 4 EPTB). The selection criteria for PTB cases was; similar to the diagnostic recommendation defined by Revised National Tuberculosis Control Program (RNTCP)^34^. In brief: was defined of a patient, presented with cough and fever >2 weeks, mysterious weight loss, fatigue, past history of patients/family members with TB or those diagnosed as having TB by the clinician and advised to receive full course of TB treatment. The medical history of the patients was obtained before collection of the blood sample and details of other investigations including chest x-ray. and other laboratory findings were recorded from the patient file. The clinically diagnosed case of TB, involving specimens from lung parenchyma or the tracheobronchial tree were classified as PTB, while clinical samples linking to organs other than the lungs, e.g. pleura, lymph nodes, abdomen, genitourinary tract, skin, joints and bones, meninges were classified as EPTB cases. The clinical isolates obtained from PTB and EPTB cases were subjected to first line drug susceptibility testing; those isolates showing resistance to isoniazid and rifampicin were classified as MDR-TB cases. Only three types of patients with HIV, Toxoplasmosis, Leishmaniasis (confirmed by commercial ICTs/ELISA based test) were included in the disease control group. The TB status in the participants of diseased group was confirmed by MGIT960 culture. Other patients with disease such as hepatitis, cancer, diabetes and other autoimmune diseases were excluded. The blood samples of all TB negative participants (healthy volunteers) were selected from the individuals with no sign/history of TB and who tested negative by tuberculin skin test (TST). The TST was performed by a trained phlebotomist. The 5 TU/0.1 mL tuberculin (Span Diagnostics, Surat, India) was administrated intradermally on the volar aspect of the forearm and read after 48–72 hours; an induration ≥10 mm was defined as positive. All serum samples were stored in different aliquots and stored at −80 °C. Other patients details such as age, gender and vaccination and are listed in Table 2.

### Western blot and dot-blot Assays

The purified proteins were resolved on 12% SDS-PAGE and transferred on to nitrocellulose membranes using semi-dry blotting apparatus (Bio-Rad, Hercules USA) following manufacturer instructions. For dot-blot, PBS was used as the spotting and dilution buffer for all purified recombinant proteins. A dot-blot was x spotted on a nitrocellulose membrane using a Bio-Dot 96-well manifold apparatus (Bio-Rad, Hercules, USA). The 100 μl of diluted proteins were loaded on each well of the assembled 96-well dot-blot apparatus with the final concentration of proteins 25 ng/well. The membrane was blocked with blocking buffer (5% skimmed milk in 1x Phosphate Buffer Saline (PBS)) for overnight at 4 °C. Membrane was washed 5 times with 1x PBST (1xPBS + 0.5% Tween20) and probed with culture confirmed PTB, EPTB or MDR-TB patients’ sera (1:200 dilutions in 1xPBS containing 0.25% bovine serum albumin) for 2 hrs at 37 °C. Serum samples from healthy and disease controls were also tested in the same manner. After Incubation, membrane was again washed 5 times with 1x PBST and probed with anti-human IgG (whole molecule) HRP-conjugated antibody (Sigma, USA) as the secondary antibody (1:8000 dilutions) for 2 hrs at 37 °C. The membranes were developed by using 3,3′-Diaminobenzidine (DAB), (Sigma Aldrich, USA) as substrate through incubation at room temp for 30–40 second as substrate for 30–40 sec at RT.

### Indirect Enzyme-Linked Immunosorbent Assay (ELISA)

Checkerboard titration (CBT) method was used for the detection of optimum serum dilution and antigen concentration in ELISA. Ninety-six well flat bottom plates (BD, New Jersey, USA) were coated with purified antigens of 25 ng/well (rSS1 and rSS5) or 50 ng/well (rSS2, rSS3, rSS4, esat6 and 38 kDa Ag) in 100 μl/well 0.1 M bicarbonate buffer (pH 9.6) and incubated at 4 °C for overnight. Plates were washed 3 times with 1x PBST, blocked with blocking buffer (5% fat free skimmed milk in PBS) for two hour at 37 °C and followed by three washing with 1x PBST. Optimum serum dilutions (1:50) were added on each well and incubated for 2 h at 37 °C. The plates were again washed three times with PBST and then incubated for 2 h with anti-human IgG, whole antibody conjugated with horseradish peroxidase (1:15,000 dilutions) followed by washing with 1x PBST. Enzyme activity was assayed by incubation for 15 min at 37 °C with 100 μl of tetramethylbenzidine (TMB) per well. Reaction was stopped by using 50 μl of 2N sulfuric acid in each well and the OD was taken at 450 nm. All the serum samples were tested in duplicate wells and experiments were repeated three times to verify the reproducibility of results. All *in-vitro* methods were performed in accordance with the standard guidelines and institutional regulations and following the manufacturer’s instructions, if a commercial equipment, device or kit was used.

### Statistical analysis

Data were presented as means and standard deviation (mean ± 2 SD). The cut-off value of an ELISA was determined by using receiver operative characteristic (ROC) curve analysis. ROC curves were plotted using STATA SE.11.0 (Stata Corp LP, Texas, USA) software. ROC curve describes probability of accuracy of the test result at different cut-off values. The individual sample was scored positive, if the OD was above the Youden index (YI) value. Sensitivity was determined by dividing the number of positive cases by the total number of TB patients. Specificity was determined by dividing the number of negative controls by the total number of healthy controls.

## Additional Information

**How to cite this article:** Singh, A. *et al*. Evaluation of 5 Novel protein biomarkers for the rapid diagnosis of pulmonary and extra-pulmonary tuberculosis: preliminary results. *Sci. Rep.* **7**, 44121; doi: 10.1038/srep44121 (2017).

**Publisher's note:** Springer Nature remains neutral with regard to jurisdictional claims in published maps and institutional affiliations.

## Supplementary Material

Supplementary Information

## Figures and Tables

**Table 1:** Vectors and primers used for cloning of the five novel proteins of *Mycobacterium tuberculosis* (Mtb) named as rSS1, rSS2, rSS3, rSS4 and rSS5.

S. No.	Gene(s)	Plasmid	Cloning vector (cloning sites)	Primers used for amplification
1	rSS1	pQE30	pQE-SS1	F 5′-CGGGATCCATGCCGCTTACACCTGCC-3′
			(*BamH*I & *Hind*III)	R 5′-CCCAAGCTTCTAGTTTTTGCCCCGGTTGAA-3′
2	rSS2	pQE30	pQE-SS2	F 5′-CGGGATCCATGACGGCAATCTCGTGCTC-3′
			(*BamH*I & *Hind*III)	R 5′-CCCAAGCTTTTAGCTGGCCGCCAGCTG-3′
3	rSS3	pQE30	pQE-SS3	F 5′-CGGGATCCATGAGCGTTGCAAACCTCAAG-3′
			(*BamH*I & *Hind*III)	R 5′-CCCAAGCTTTCACAAAACTCCTCCGGTTGG-3′
4	rSS4	pQE30	pQE-SS4	F 5′-CGGGATCCGTG ACG GAC ATG AAC CCG GA-3′
			(*BamH*I & *Hind*III)	R 5′-CCCAAGCTTTCA CGG GCC CCC GGT ACT-3′
5	rSS5	pQE30	pQE30-SS5	F 5′-CGGGATCCATGACCAAGAGTCTGCCAGG-3
			(*BamH*I & *Hind*III)	R 5′-CCCAAGCTT^#^TCAAACCCGGCTAAGGTGC-3

Nucleotide sequences recognised by the restriction enzyme used for cloning are underlined.

**Table 2:** Detailed clinical and demographical profile of subjects (N = 250) included in the study.

S. No.	Category (n)	Mean Age (Yr) ± SD	Gender		HIV status		BCG Vaccination		Mantoux test		Smear		MGIT Culture (%)	MDR (%)
			Male (%)	Female (%)	Pos (%)	Neg (%)	Yes (%)	No/uk (%)	Pos (%)	Neg/uk (%)	Pos (%)	Neg (%)		
1	PTB (n = 111)	35.0 ± 15.2	63 (56.8)	48 (43.2)	15 (13.5)	96 (86.5)	42 (37.8)	69 (62.2)	68 (61.3)	43 (38.7)	72 (64.9)	39 (35.1)	111 (100)	46 (41.4)
2	EPTB (n = 29)	31.0 ± 16.1	15 (51.7)	14 (48.3)	5 (17.2)	24 (82.8)	10 (34.5)	19 (65.5)	11 (37.9)	18 (62.1)	4 (13.8)	25 (86.2)	29 (100)	4 (13.8)
4	HC (n = 50)	30.0 ± 13.2	30 (60.0)	20 (40.0)	0	50 (100)	14 (28.0)	36 (72.0)	—	50 (100)	0	50 (100)	0	0
5	DC (non-TB) n = 60	31.4 ± 14.6	34 (56.7)	26 (43.3)	20 (33.3)	40 (66.7)	18 (30.0)	42 (70.0)	—	60 (100)	0	60 (100)	0	0
**Total**	**250**	**31.8 ± 14.8**	**142 (56.8)**	**108 (43.2)**	**40 (16.0)**	**210 (84.0)**	**84 (33.6)**	**166 (66.4)**	**79 (31.6)**	**171 (68.4)**	**76 (30.4)**	**174 (69.6)**	**140 (56.0)**	**50 (20.0)**

**Table 3:** Sensitivity and specificity of 5 novel recombinant antigens by dot-blot assay [PTB = 111, EPTB = 29 and controls = 110].

	Sensitivity [n (%; 95% CI)]	Specificity [n (%; 95% CI)]	PPV (%)	NPV (%)	LRP (95% CI)	DA %, (95% CI)
PTB cases (n = 111)						
rSS5 (Rv2970c)	*110/111 (99.1%; 95.1, 99.8)*	*108/110 (98.2%; 93.6, 99.6)*	*98.2%*	*99.1%*	54.5 (20.4–145.2)	98.6% (96.1, 99.5)
rSS1 (Rv2145c)	*110/111 (99.1%; 95.1, 99.8)*	*110/110 (100%; 96.6, 100)*	*100%*	*99.1%*	*−*	*99.5% (97.5, 99.9)*
rSS4 (Rv1827)	*109/111 (98.2%; 93.7, 99.5)*	*103/110 (93.6%; 87.4, 96.9)*	*94.0%*	*98.1%*	*15.4 (11.6–20.4)*	*95.9% (92.4, 97.8)*
rSS2 (Rv0164)	*107/111 (96.4%; 91.1, 98.6)*	*102/110 (92.7%; 86.3, 96.3)*	*93.0%*	*96.2%*	*13.2 (10.4–16.9)*	*94.6% (90.7, 96.9)*
rSS3 (Rv1437)	*104/111 (93.7%; 87.5, 96.9)*	*98/110 (89.1%; 81.9, 93.6)*	*89.7%*	*93.3%*	*8.6 (7.3–10.1)*	*91.4% (87.0, 94.4)*
Esat6 Ag	*95/111 (85.6%; 77.9, 90.9)*	*73/110 (66.4%; 57.1, 74.51)*	*72.0%*	*82.0%*	*2.5 (2.4–2.7)*	*76.0% (70.0, 81.2)*
38 kDa Ag	*95/11116 (85.6%; 77.9, 90.9)*	*60/110 (54.5%; 45.2, 63.5)*	*65.5%*	*78.9%*	*1.9 (1.8–2.0)*	*70.1% (63.8, 75.8)*
EPTB cases (n = 29)						
rSS5 (Rv2970c)	*28/29 (96.5%; 82.8, 99.4)*	*108/110 (98.2%; 93.6, 99.6)*	*93.3%*	*99.1%*	*53.1 (19.9–141.8)*	*97.8% (93.8, 99.3)*
rSS1 (Rv2145c)	*27/29 (93.1%; 78.0, 98.1)*	*110/110 (100%; 96.6, 100)*	*100%*	*98.2%*	*—*	*98.6% (94.9, 99.6)*
rSS4 (Rv1827)	*27/29 (93.1%; 78.0, 98.1)*	*103/110 (93.6%; 87.4, 96.9)*	*79.4%*	*98.1%*	*14.6 (11.0–19.5)*	*93.5% (88.1, 96.6)*
rSS2 (Rv0164)	*25/29 (86.2%; 69.4, 94.5)*	*102/110 (92.7%; 86.3, 96.3)*	*75.8%*	*96.2%*	*11.8 (9.2–15.3)*	*91.4% (85.5, 95.0)*
rSS3 (Rv1437)	*26/29 (89.7%; 73.6, 96.4)*	*98/110 (89.1%; 81.9, 93.6)*	*68.4%*	*97.0%*	*8.2 (6.9–9.8)*	*89.2% (83.0, 93.3)*
Esat6 Ag	*21/29 (72.4%; 54.3, 85.3)*	*73/110 (66.4%; 57.1, 74.51)*	*36.2%*	*90.1%*	*2.1 (2.0–2.3)*	*67.6 (59.5–74.8)*
38 kDa Ag	*25/29 (86.2%; 69.4, 94.5)*	*60/110 (54.5%; 45.2, 63.5)*	*33.4%*	*93.7%*	*2.0 (1.8–2.0)*	*61.1% (52.8, 68.8)*
MDR-TB Cases [(n = 50)						
rSS5 (Rv2970c)	*50/50 (100%; 92.9, 100)*	*108/110 (98.2%; 93.6, 99.6)*	*96.1%*	*100%*	*55.0 (20.6–146.5)*	*98.7% (95.6, 99.7)*
rSS1 (Rv2145c)	*50/50 (100%; 92.9, 100)*	*110/110 (100%; 96.6, 100)*	*100%*	*100%*	*—*	*100% (97.7, 100)*
rSS4 (Rv1827)	*50/50 (100%; 92.9, 100)*	*103/110 (93.6%; 87.4, 96.9)*	*87.7%*	*100%*	*15.7 (11.9–20.8)*	*95.6% (91.2, 97.9)*
rSS2 (Rv0164)	*50/50 (100%; 92.9, 100)*	*102/110 (92.7%; 86.3, 96.3)*	*86.2%*	*100%*	*13.7 (10.8–17.6)*	*95.0% (90.4, 97.4)*
rSS3 (Rv1437)	*50/50 (100%; 92.9, 100)*	*98/110 (89.1%; 81.9, 93.6)*	*80.6%*	*100%*	*9.2 (7.8–10.8)*	*92.5% (87.3, 95.7)*
Esat6 Ag	*45/50 (90.0%;78.6, 95.6)*	*73/110 (66.4%; 57.1, 74.51)*	*54.9%*	*93.6%*	*2.7 (2.5–2.8)*	*73.7% (66.4, 79.9)*
38 kDa Ag	*47/50 (94.0%;83.8, 97.9)*	*60/110 (54.5%; 45.2, 63.5)*	*48.4%*	*95.2%*	*2.1 (2.0–2.2)*	*66.9% (59.3, 73.7)*

Pos: Positive, Neg: Negative, CI: Confidence interval, PPV: Positive predictive value, NPV: Negative predictive value, LRP: likelihood ratio for positive test, DA: Diagnostic accuracy. *Also see supplementary file 1.

**Table 4:** Sensitivity and specificity of 5 novel recombinant antigens by ELISA test [PTB = 111, EPTB = 29 and control = 110].

	Sensitivity [n (%; 95% CI)]	Specificity [n (%; 95% CI)]	PPV (%)	NPV (%)	LRP (95% CI)	DA%, (95% CI)
PTB cases (n = 111)						
rSS5 (Rv2970c)	*107/111 (96.4%; 91.1, 98.6)*	*108/110 (98.2%; 93.6, 99.5)*	*98.2%*	*96.4%*	*53.2 (19.9–141.4)*	*97.3% (94.2, 98.7)*
rSS1 (Rv2145c)	*107/111 (96.4%; 91.1, 98.6)*	*107/110 (97.3%; 92.3, 99.1)*	*97.3%*	*96.4%*	*35.3 (18.4–68.0)*	*96.8% (93.6, 98.5)*
rSS4 (Rv1827)	*102/111 (91.9%; 85.3, 95.7)*	*104/110 (94.5%; 88.6, 97.5)*	*94.5%*	*92.0%*	*16.8 (12.1–23.4)*	*93.2% (89.1, 95.8)*
rSS2 (Rv0164)	*81/111 (73.0%; 64.0, 80.4)*	*96/110 (87.3%; 79.8, 92.3)*	*85.3%*	*76.2%*	*5.7 (4.9–6.6)*	*80.1% (74.3, 84.8)*
rSS3 (Rv1437)	*82/111 (73.9%; 65.0, 81.1)*	*95/110 (86.7%; 78.7, 91.6)*	*84.5%*	*76.6%*	*5.4 (4.7–6.2)*	*80.1% (74.3, 84.8)*
Esat6 Ag	*81/111 (72.97%;64.0, 80.4)*	*78/110 (70.9%; 61.8, 78.6)*	*71.7%*	*72.2*	*2.5 (2.3–2.7)*	*71.9% (65.7–77.5)*
38 kDa Ag	*103/11 (92.8%;86.4, 96.3)*	*67/110 (60.9%; 51.6, 69.5)*	*70.5%*	*89.3%*	*2.4 (2.3–2.5)*	*76.9% (70.9, 82.0)*
EPTB cases (n = 29)						
rSS5 (Rv2970c)	*28/29 (96.5%; 82.8, 99.4)*	*108/110 (98.2%; 93.6, 99.5)*	*93.3%*	*99.1%*	*53.1 (19.9–141.8)*	*97.8% (93.8, 99.3)*
rSS1 (Rv2145c)	*26/29 (89.7%; 73.6, 96.4)*	*107/110 (97.3%; 92.3, 99.1)*	*89.7%*	*97.3%*	*32.9 (17.0–63.7)*	*95.7% (90.9, 98.0)*
rSS4 (Rv1827)	*26/29 (89.7%; 73.6, 96.4)*	*104/110(94.5%; 88.6, 97.5)*	*81.2%*	*97.2%*	*16.4 (11.7–23.0)*	*93.5% (88.1, 96.6)*
rSS2 (Rv0164)	*20/29 (69.0%; 50.8, 82.7)*	*96/110 (87.3%; 79.8, 92.3)*	*58.8%*	*91.4%*	*5.4 (4.5–6.5)*	*83.4% (76.4, 88.7)*
rSS3 (Rv1437)	*22/29 (75.9%; 57.9, 87.8)*	*95/110 (86.7%; 78.7, 91.6)*	*59.5%*	*93.1%*	*5.6 (4.7–6.5)*	*84.2% (77.2, 89.3)*
Esat6 Ag	*26/29 (89.7%; 73.6, 96.4)*	*78/110 (70.9%; 61.8, 78.6)*	*44.8%*	*96.3%*	*3.1 (2.9–3.3)*	*74.8% (67.8, 81.3)*
38 kDa Ag	*26/29 (89.7%; 73.6, 96.4)*	*67/110 (60.9%; 51.6, 69.5)*	*37.7%*	*95.7%*	*2.3 (2.2–2.4)*	*66.9% (58.7, 74.2)*
MDR-TB Cases [(n = 50)						
rSS5 (Rv2970c)	*50/50 (100%; 92.9, 100)*	*108/110 (98.2%; 93.6, 99.5)*	*96.1%*	*100%*	*55.0 (20.6–146.5)*	*98.7% (95.6, 99.6)*
rSS1 (Rv2145c)	*50/50 (100%; 92.9, 100)*	*107/110 (97.3%; 92.3, 99.1)*	*94.3%*	*100%*	*37.7 (19.1–70.5)*	*98.1% (94.6, 99.4)*
rSS4 (Rv1827)	*50/50 (100%; 92.9, 100)*	*104/110(94.5%; 88.6, 97.5)*	*89.3%*	*100%*	*18.3 (13.2–25.4)*	*96.2% (92.1, 98.3)*
rSS2 (Rv0164)	*38/50 (76.0%; 62.6, 85.7)*	*96/110 (87.3%; 79.8, 92.3)*	*73.1%*	*88.9%*	*6.0 (5.1–7.0)*	*83.7% (77.2, 88.7)*
rSS3 (Rv1437)	*41/50 (82.0%; 69.2, 90.2)*	*95/110 (86.7%; 78.7, 91.6)*	*73.2%*	*91.3%*	*6.0 (5.2–6.9)*	*85.0% (78.6, 89.7)*
Esat6 Ag	*33/50 (66.0%; 52.1, 77.6)*	*78/110 (70.9%; 61.8, 78.6)*	*50.8%*	*82.1%*	*2.3 (2.1–2.5)*	*69.4% (61.8, 76.0)*
38 kDa Ag	*47/50 (94.0%; 83.8, 97.9)*	*67/110 (60.9%; 51.6, 69.5)*	*55.2%*	*95.7%*	*2.4 (2.3–2.5)*	*71.2% (63.8, 77.7)*

Pos: Positive, Neg: Negative, CI: Confidence interval, PPV: Positive predictive value, NPV: Negative predictive value, LRP: likelihood ratio for positive test, DA: Diagnostic accuracy. *Also see supplementary file 1.
