# Supplementary material for: Evaluation of 5 Novel protein biomarkers for the rapid diagnosis of pulmonary and extra-pulmonary tuberculosis: preliminary results
Source: Sci Rep. 2017 Mar 24;7:44121. doi: 10.1038/srep44121 (PMC5364505; doi:10.1038/srep44121)
Supplement: Supplementary Information [file srep44121-s1.doc]

**Evaluation of 5 Novel protein biomarkers for the rapid diagnosis of pulmonary and extra-pulmonary tuberculosis: preliminary results**

Amit Singh^1$^, Anil Kumar Gupta^1$^, Krishnamoorthy Gopinath^1,#^, Pawan Sharma^2,¶^, Sarman Singh^1,*^

^1^Division of Clinical Microbiology & Molecular Medicine, All India Institute of Medical Sciences, New Delhi; ^2^International Centre for Genetic Engineering and Biotechnology, New Delhi

**Running Title: Novel recombinant antigens for TB diagnosis**

^#^Present Address: Max Planck Institute for Infectious Biology, Charitéplatz, Berlin, Germany

^¶^ North East Region-BPMC, Department of Biotechnology, Government of India, New Delhi

***Corresponding Author**

Prof. Sarman Singh,

All India Institute of Medical Sciences,

Ansari Nagar, New Delhi-110 029, India

Phone: +91-11-26588484,

Fax: +91-11-26588641

Email: [sarman_singh@yahoo.com](mailto:sarman_singh@yahoo.com)

**Supplementary Table 1: Comparison of dot-blot sensitivity and specificity of *M. tuberculosis*** recombinant antigens in bacteriologically confirmed PTB cases (n=111)

| **Antigens/group** | **Sensitivity [n (%; 95% CI)]** | **Specificity [n (%; 95% CI)]** | **PPV (%)** | **NPV (%)** | **LRP (95% CI)** | **DA %, (95%CI)** |
| --- | --- | --- | --- | --- | --- | --- |
| **Rv2970c (lipN)** | | | | | | |
| *PTB cases (n=111)* | *110/111 (99.1%; 95.1, 99.8)* | *108/110 (98.2%;93.6,99.6)* | *98.2%* | *99.1%* | 54.5 (20.4-145.2) | 98.6% (96.1, 99.5) |
| HIV Pos (n=15) | 15/15 (100%; 79.6, 100) | 108/110 (98.2%;93.6,99.6) | 88.2% | 100% | 55.0 (20.6-146.5) | 98.4% (94.4, 99.6) |
| HIV Neg (n=96) | 95/96 (99.0%; 94.3, 99.8) |  | 97.9% | 99.1% | 54.4 (20.4-145.1) | 98.5% (95.8, 99.5) |
| Mx pos (n=68) | 68/68 (100%; 94.6, 100) |  | 97.1% | 100% | 55.0 (20.6-146.5) | 98.9% (96.1, 99.7) |
| Mx Neg/ND (n=43) | 42/43 (97.7%; 88.2, 99.6) |  | 95.4% | 99.1% | 53.7 (20.1-143.3) | 98.0% (94.4, 99.3) |
| BCG vacc. (n=42) | 42/42 (100%; 91.6, 100) |  | 95.4% | 100% | 55.0 (20.6-146.5) | 98.7% (95.3, 99.6) |
| BCG uk/non vacc. (n=69) | 68/69 (96.5%; 82.8, 99.4) |  | 93.3% | 99.1% | 53.1 (19.9-141.8) | 97.8% (93.8, 99.3) |
| S (+), C (+), n=58 | 58/58 (100%; 91.6, 100) |  | 95.4% | 100% | 55.0 (20.6-146.5) | 98.7% (96.6, 99.6) |
| S (-), C (+), n=53 | 52/53 (98.1%; 90.1, 99.7) |  | 96.3% | 99.1% | 54.0 (20.2-143.9) | 98.2% (94.7, 99.4) |
| **Rv2145c (wag31)** | | | | | | |
| *PTB cases (n=111)* | *110/111 (99.1%; 95.1, 99.8)* | *110/110 (100%;96.6,100)* | *100%* | *99.1%* | *-* | *99.5% (97.5, 99.9)* |
| HIV Pos (n=15) | 15/15 (100%; 79.6, 100) | 110/110 (100%;96.6,100) | 100% | 100% | - | 100% (97.0, 100) |
| HIV Neg (n=96) | 95/96 (99.0%; 94.3, 99.8) |  | 100% | 99.1% | - | 99.5% (97.3, 99.8) |
| Mx pos (n=68) | 68/68 (100%; 94.6, 100) |  | 100% | 100% | - | 100% (97.9, 100) |
| Mx Neg/ND (n=43) | 42/43 (97.7%; 88.2, 99.6) |  | 100% | 99.1% | - | 99.3% (96.4, 99.9) |
| BCG vacc. (n=42) | 42/42 (100%; 91.6, 100) |  | 100% | 100% | - | 100% (97.5, 100) |
| BCG uk/no vacc. (n=69) | 68/69 (98.5%; 92.2, 99.7) |  | 100% | 99.1% | - | 99.4% (96.9, 99.9) |
| S (+), C (+), n=58 | 58/58 (100%; 75.7, 100) |  | 100% | 100% | - | 100% (96.9, 100) |
| S (-), C (+), n=53 | 52/53 (98.1%; 90.1, 99.7) |  | 100% | 99.1% | - | 99.4% (96.6, 99.9) |
| **Rv1827 (garA)** | | | | | | |
| *PTB cases (n=111)* | *109/111(98.2%; 93.7, 99.5)* | *103/110 (93.6%;87.4, 96.9)* | *94.0%* | *98.1%* | *15.4 (11.6-20.4)* | *95.9% (92.4, 97.8)* |
| HIV Pos (n=15) | 15/15 (100%; 79.6, 100) | 103/110 (93.6%;87.4, 96.9) | 68.2% | 100% | 15.7 (11.9-20.8) | 94.4% (88.9, 97.3) |
| HIV Neg (n=96) | 94/96 (92.7%; 85.7, 96.4) |  | 93.7% | 93.7% | 17.0 (12.2-23.6) | 93.7% (89.5, 96.3) |
| Mx pos (n=68) | 68/68 (100%; 94.6, 100) |  | 90.7% | 100% | 15.7 (11.9-20.8) | 96.1% (92.1, 98.1) |
| Mx Neg/ND (n=43) | 41/43 (95.3%; 84.5, 98.7) |  | 85.4% | 98.1% | 15.0 (11.3-19.9) | 94.1% (89.2, 96.8) |
| BCG vacc. (n=42) | 42/42 (100%; 91.6, 100) |  | 85.7% | 100% | 15.7 (11.9-20.8) | 95.4% (90.8, 97.7) |
| BCG uk/no vacc. (n=69) | 67/69 (97.1%; 90.0, 99.2) |  | 90.5% | 98.1% | 15.3 (11.5-20.2) | 95.0% (90.7, 97.3) |
| S (+), C (+), n=58 | 58/58 (100%; 75.7, 100) |  | 63.2% | 100% | 15.7 (11.9-20.8) | 94.3% (88.6, 97.2) |
| S (-), C (+), n=53 | 51/53 (96.2%; 87.2, 99.0) |  | 94.5% | 98.2% | 35.3 (18.3-67.9) | 96.9% (93.0, 98.7) |
| **Contd... Supplementary Table 1** | | | | | | |
| **Antigens/group** | **Sensitivity [n (%; 95% CI)]** | **Specificity [n (%; 95% CI)]** | **PPV (%)** | **NPV (%)** | **LRP (95% CI)** | **DA %, (95%CI)** |
| **Rv0164 (TB 18.50** | | | | | | |
| *PTB cases (n=111)* | *107/111(96.4%; 91.1, 98.6)* | *102/110 (92.7%;86.3, 96.3)* | *93.0%* | *96.2%* | *13.2 (10.4-16.9)* | *94.6% (90.7, 96.9)* |
| HIV Pos (n=15) | 14/15 (93.3%; 70.2, 98.8) | 102/110 (92.7%;86.3, 96.3) | 63.6% | 99.0% | 12.8 (9.9-16.6) | 92.8% (86.9, 96.2) |
| HIV Neg (n=96) | 93/96 (96.9%; 91.2, 98.9) |  | 92.1% | 97.1% | 13.3 (10.4-14.0) | 94.7% (90.7, 97.0) |
| Mx pos (n=68) | 68/68 (100%; 94.6, 100) |  | 89.5% | 100% | 13.7 (10.8-17.6) | 95.5% (91.4, 97.7) |
| Mx Neg/ND (n=43) | 39/43 (90.7%; 78.4, 96.3) |  | 83.0% | 96.2% | 12.5 (9.7-16.0) | 92.2% (86.8, 95.5) |
| BCG vacc. (n=42) | 42/42 (100%; 91.6, 100) |  | 84.0% | 100% | 13.7 (10.8-17.6) | 90.0% (90.0, 97.3) |
| BCG uk/no vacc. (n=69) | 65/69 (94.2%; 86.0, 97.7) |  | 89.0% | 96.2% | 12.9 (10.1-16.6) | 93.3% (88.6, 96.1) |
| S (+), C (+), n=58 | 57/58 (98.2%; 90.9, 99.7) |  | 87.7% | 99.0% | 13.5 (10.6-17.3) | 94.6% (90.1, 97.2) |
| S (-), C (+), n=53 | 50/53 (94.3%; 84.6, 98.1) |  | 86.2% | 97.1% | 13.0 (10.1-16.6) | 93.2% (88.3, 96.2) |
| **Rv1437 (pgk)** | | | | | | |
| *PTB cases (n=111)* | *104/111(93.7%; 87.5, 96.9)* | *98/110 (89.1%;81.9, 93.6)* | *89.7%* | *93.3%* | *8.6 (7.3-10.1)* | *91.4% (87.0, 94.4)* |
| HIV Pos (n=15) | 13/15 (86.7%; 62.1, 96.3) | 98/110 (89.1%;81.9, 93.6) | 52.0% | 98.0% | 7.9 (6.6-9.6) | 88.8% (82.1, 93.2) |
| HIV Neg (n=96) | 91/96 (94.8%; 88.4, 97.8) |  | 88.3% | 95.1% | 6.7 (7.4-10.2) | 91.7% (87.2, 94.8) |
| Mx pos (n=68) | 66/68 (97.1%; 89.9, 99.2) |  | 84.6% | 98.0% | 8.9 (7.5-10.5) | 92.1% (87.2, 95.3) |
| Mx Neg/ND (n=43) | 38/43 (88.3%; 75.5, 94.9) |  | 76.0% | 95.1% | 8.1 (6.8-9.6) | 88.9% (82.9, 92.9) |
| BCG vacc. (n=42) | 40/42 (95.2%; 84.2, 98.7) |  | 76.9% | 98.0% | 8.7 (7.4-10.3) | 90.8% (85.1, 94.4) |
| BCG uk/no vacc. (n=69) | 64/69 (92.7%; 84.1, 96.9) |  | 84.2% | 95.1% | 8.5 (7.2-10.0) | 90.5% (85.3, 94.0) |
| S (+), C (+), n=58 | 56/58 (96.55%; 88.3, 99.0) |  | 82.3% | 98.0% | 8.8 (7.5-10.3) | 91.7% (86.5, 95.0) |
| S (-), C (+), n=53 | 47/53 (88.7%; 77.4, 94.7) |  | 79.7% | 94.2% | 8.1 (6.9-9.6) | 89.0% (83.2, 92.9) |
| **Esat6 Ag** | | | | | | |
| *PTB cases (n=111)* | *95/111 (85.6%;77.9, 90.9 )* | *73/110 (66.4%;57.1, 74.51)* | *72.0%* | *82.0%* | *2.5(2.4-2.7)* | *76.0% (70.0, 81.2)* |
| HIV Pos (n=15) | 11/15 (73.33%; 48.0, 89.1) | 73/110 (66.4%;57.1, 74.51) | 22.9% | 94.8% | 2.2 (1.9-2.4) | 67.2% (58.6, 74.8) |
| HIV Neg (n=96) | 84/96 (87.5%; 79.4, 92.7) |  | 69.4% | 85.9% | 2.6 (2.5-2.7) | 76.2% (69.9, 81.5) |
| Mx pos (n=68) | 62/68 (91.18%; 82.1, 95.9) |  | 92.4% | 92.4% | 2.7 (2.6-2.9) | 75.8% (69.0, 81.5) |
| Mx Neg/ND (n=43) | 33/43 (6.74%; 62.3, 86.8) |  | 47.1% | 87.9% | 2.3 (2.1-2.4) | 69.3% (61.6, 76.0) |
| BCG vacc. (n=42) | 35/42 (83.3%; 69.4, 9.7) |  | 32.4% | 84.1% | 1.3 (1.2-1.3) | 47.4% (39.6, 55.3) |
| BCG uk/no vacc. (n=69) | 60/69 (86.96%; 77.0, 93.0) |  | 45.1% | 80.4% | 1.3 (1.3-1.4) | 54.2% (46.9, 61.3) |
| S (+), C (+), n=58 | 53/58 (91.4%; 81.4, 96.4) |  | 42.1% | 88.1% | 1.4 (1.3-1.4) | 53.6% (46.0, 61.0) |
| S (-), C (+), n=53 | 42/53 (79.25%; 66.5, 88.0) |  | 36.5% | 77.1% | 1.2 (1.1-1.2) | 48.5% (40.9, 56.1) |
| **Contd... Supplementary Table 1** | | | | | | |
| **Antigens/group** | **Sensitivity [n (%; 95% CI)]** | **Specificity [n (%; 95% CI)]** | **PPV (%)** | **NPV (%)** | **LRP (95% CI)** | **DA %, (95%CI)** |
| **38kDa Ag** | | | | | | |
| *PTB cases (n=111)* | *95/111(85.6%; 77.9, 90.9)* | *60/110 (54.5%;45.2, 63.5)* | *65.5%* | *78.9%* | *1.9 (1.8-2.0)* | *65.6% (59.1, 71.6)* |
| HIV Pos (n=15) | 11/15 (73.3%; 48.0, 89.) | 60/110 (54.5%;45.2, 63.5) | 18.0% | 93.7% | 1.6 (1.4-1.8) | 56.8% (48.0, 65.1) |
| HIV Neg (n=96) | 84/96 (87.5%; 79.4, 92.7) |  | 54.2% | 73.1% | 1.9 (1.8-2.0) | 69.9% (63.3, 75.8) |
| Mx pos (n=68) | 60/68 (88.2%; 78.5, 93.9) |  | 54.5% | 88.2% | 1.9 (1.9-2.0) | 67.4% (60.2, 73.9) |
| Mx Neg/ND (n=43) | 35/43 (81.4%; 67.4, 90.3 ) |  | 41.2% | 8.2% | 1.8 (1.7-1.9) | 62.1% (54.2, 69.4) |
| BCG vacc. (n=42) | 36/42 (85.7%; 72.2, 93.3) |  | 41.9% | 90.9% | 1.9 (1.8-2.0) | 63.2% (55.2, 70.4) |
| BCG uk/no vacc. (n=69) | 59/69 (85.5%; 75.3, 91.9) |  | 54.1% | 85.7% | 1.9 (1.8-2.0) | 66.5% (59.3. 73.0) |
| S (+), C (+), n=58 | 54/58 (93.1%; 83.6, 97.3) |  | 51.9% | 93.7% | 2.0 (2.0, 2.1) | 67.9% (60.5, 74.4) |
| S (-), C (+), n=53 | 41/53 (77.4%; 64.5, 86.5) |  | 45.0% | 83.3% | 1.7 (1.6-1.8) | 62.0% (54.3, 69.1) |
| Pos: Positive, Neg: Negative, CI: Confidence interval, PPV: Positive predictive value, NPV: Negative predictive value, LRP: likelihood ratio for positive test, DA: Diagnostic accuracy, uk-unknown, Mx: Mantoux test, ND: not done, Vacc:-vaccination. C: MGIT 960 culture, S: ZN stained smear, *MDR-TB cases are DST confirmed 46 from PTB and 4 from EPTB group. | | | | | | |

**Supplementary Table 2: Comparison of dot-blot sensitivity and specificity of *M. tuberculosis*** recombinant antigens in bacteriologically confirmed EPTB cases (n=29)

|  | **Sensitivity [n (%; 95% CI)]** | **Specificity [n (%; 95% CI)]** | **PPV (%)** | **NPV (%)** | **LRP (95% CI)** | **DA %, (95%CI)** |
| --- | --- | --- | --- | --- | --- | --- |
| **Rv2970c (lipN)** | | | | | | |
| *EPTB cases (n=29)* | *28/29 (96.5%; 82.8, 99.4)* | *108/110 (98.2%;93.6,99.6)* | *93.3%* | *99.1%* | *53.1 (19.9-141.8)* | *97.8% (93.8, 99.3)* |
| HIV Pos (n=5) | 5/5 (100%; 75.7, 100) | 108/110 (98.2%;93.6,99.6) | 85.7% | 100% | 55.0 (20.6-146.5) | 98.4% (94.2, 99.5) |
| HIV Neg (n=24) | 23/24 (99.0%; 94.5, 99.8) |  | 98.2% | 99.1% | 54.4 (20.4-145.1) | 98.6% (95.9, 99.5) |
| Mx pos (n=8) | 8/8 (100%; 56.5, 100) |  | 71.4% | 100% | 55.0 (20.6-146.5) | 98.3% (93.9, 99.5) |
| Mx Neg/ND (n=21) | 20/21 (95.2%; 79.8, 99.3) |  | 90.9% | 99.1% | 52.3 (19.5-140.3) | 97.7% (94.5, 99.8) |
| BCG vacc. (n=10) | 10/10 (100%; 72.2, 100) |  | 93.3% | 100% | 55.0 (20.6-146.5) | 98.3% (94.1, 99.5) |
| BCG uk/no vacc. (n=19) | 18/19 (95.0%; 76.4, 99.1) |  | 90.5% | 99.1% | 52.2 (19.5-140) | 97.7% (93.4, 99.2) |
| S (+), C (+), n=4 | 4/4 (100%; 51.0, 100) |  | 66.7% | 100% | 55.0 (20.6-146.5) | 98.2% (93.8, 99.5) |
| S (-), C (+), n=25 | 24/25 (96.0%; 80.5, 99.3) |  | 92.3% | 99.1% | 52.8 (19.7-141.2) | 97.8% (93.7, 99.2) |
| **Rv2145c (wag31)** | | | | | | |
| *EPTB cases (n=29)* | *27/29 (93.1%; 78.0, 98.1)* | *110/110 (100%;96.6,100)* | *100%* | *98.2%* | *-* | *98.6% (94.9, 99.6)* |
| HIV Pos (n=5) | 5/5 (100%; 56.5, 100) | 110/110 (100%;96.6,100) | 100% | 100% | - | 100% (96.8, 100) |
| HIV Neg (n=24) | 22/24 (91.7%; 74.1, 97.7) |  | 100% | 98.2% | - | 98.5% (94.7, 99.6) |
| Mx pos (n=8) | 8/8 (100%; 67.6, 100) |  | 100% | 100% | - | 98.3% (94.0, 99.5) |
| Mx Neg/ND (n=21) | 19/21 (95.2%; 77.3, 99.1) |  | 100% | 100% | - | 100% (96.8, 100) |
| BCG vacc. (n=10) | 9/10 (90.0%; 59.6, 98.2) |  | 100% | 99.1% | - | 99.2% (95.4, 99.8) |
| BCG uk/no vacc. (n=19) | 18/19 (94.7%; 75.4, 99.1) |  | 100% | 99.1% | - | 99.2% (95.7, 99.9) |
| S (+), C (+), n=4 | 4/4 (100%; 51.0, 100) |  | 100% | 100% | - | 100% (96.7, 100) |
| S (-), C (+), n=25 | 23/25 (92%; 75.0, 97.8) |  | 100% | 98.2% | - | 98.5% (94.8, 99.6) |
| **Rv1827 (garA)** | | | | | | |
| *EPTB cases (n=29)* | *27/29 (93.1%; 78.0, 98.1)* | *103/110 (93.6%;87.4, 96.9)* | *79.4%* | *98.1%* | *14.6 (11.0-19.5)* | *93.5% (88.1, 96.6)* |
| HIV Pos (n=5) | 5/5 (100%; 56.5, 100) | 103/110 (93.6%;87.4, 96.9) | 41.7% | 100% | 15.7 (11.9-20.8) | 93.9% (88.0, 97.0) |
| HIV Neg (n=24) | 22/24 (91.7%; 74.1, 97.7) |  | 75.9% | 98.1% | 14.4 (10.8-19.2) | 93.3% (87.7, 96.4) |
| Mx pos (n=8) | 8/8 (100%; 67.6, 100) |  | 53.3% | 100% | 15.7 (11.9-20.8) | 94.1% (88.3, 97.1) |
| Mx Neg/ND (n=21) | 19/21 (90.5%; 71.1, 97.3) |  | 73.1% | 98.1% | 14.2 (10.6-29.0) | 93.1% (87.5, 96.3) |
| BCG vacc. (n=10) | 9/10 (90.0%; 59.6, 98.2) |  | 56.2% | 99.0% | 14.1 (10.4-19.2) | 93.3% (87.4, 96.6) |
| BCG uk/no vacc. (n=19) | 18/19 (94.7%; 75.4, 99.1) |  | 72.0% | 99.0% | 15.0 (11.2-19.8) | 93.8% (88.2, 96.8) |
| S (+), C (+), n=4 | 4/4 (100%; 51.0, 100) |  | 36.4% | 100% | 15.7 (11.9-20.8) | 93.9% (87.9, 97.0) |
| S (-), C (+), n=25 | 23/25 (92.0%; 75.0, 97.8) |  | 76.7% | 98.1% | 14.5 (10.5-19.3) | 93.3% (87.8, 96.4) |
| **Contd… Supplementary Table 2** | | | | | | |
| **Antigens/group** | **Sensitivity [n (%; 95% CI)]** | **Specificity [n (%; 95% CI)]** | **PPV (%)** | **NPV (%)** | **LRP (95% CI)** | **DA %, (95%CI)** |
| **Rv0164 (TB 18.5)** | | | | | | |
| *EPTB cases (n=29)* | *25/29(86.2%; 69.4, 94.5)* | *102/110 (92.7%;86.3, 96.3)* | *75.8%* | *96.2%* | *11.8 (9.2-15.3)* | *91.4% (85.5, 95.0)* |
| HIV Pos (n=5) | 5/5 (100%; 56.5, 100) | 102/110 (92.7%;86.3, 96.3) | 38.5% | 100% | 13.7 (10.8-17.6) | 93.0% (86.9, 96.4) |
| HIV Neg (n=24) | 21/24 (87.5%; 69.0, 95.7) |  | 72.4% | 97.1% | 12.0 (9.3-15.6) | 91.8% (85.9, 95.3) |
| Mx pos (n=8) | 7/8 (87.5%; 52.9, 97.8) |  | 46.7% | 99.0% | 12.0 (9.0-16.0) | 92.4% (86.1, 95.9) |
| Mx Neg/ND (n=21) | 17/21 (80.9%; 60.9, 92.3) |  | 68.0% | 96.2% | 11.1 (8.5-14.6) | 90.8% (84.7, 94.7) |
| BCG vacc. (n=10) | 8/10 (80.0% (49.0, 94.3) |  | 50.0% | 98.1% | 11.0 (8.1-14.9) | 91.7% (85.3, 95.4) |
| BCG uk/no vacc. (n=19) | 17/19 (89.5%; 68.6, 97.1) |  | 68.0% | 98.1% | 12.3 (9.5-15.9) | 92.2% (86.3, 95.7) |
| S (+), C (+), n=4 | 3/4 (75.0%; 30.1, 95.4) |  | 27.3% | 92.1% | 10.3 (6.5-16.4) | 92.1% (85.7, 95.8) |
| S (-), C (+), n=25 | 21/25 (84.0%; 65.3, 93.6) |  | 72.4% | 96.2% | 11.5 (8.9-15.0) | 91.1% (85.1, 94.8) |
| **Rv1437 (pgk)** | | | | | | |
| *EPTB cases (n=29)* | *26/29 (89.7%; 73.6, 96.4)* | *98/110 (89.1%;81.9, 93.6)* | *68.4%* | *97.0%* | *8.2 (6.9-9.8)* | *89.2% (83.0, 93.3)* |
| HIV Pos (n=5) | 5/5 (100%; 56.5, 100) | 98/110 (89.1%;81.9, 93.6) | 29.4% | 100% | 9.2 (7.8-10.8) | 89.6% (82.6, 93.9) |
| HIV Neg (n=24) | 21/24 (87.5%; 69.0, 95.7) |  | 63.6% | 97.0% | 8.0 (6.7-9.6) | 88.8% (82.3, 93.1) |
| Mx pos (n=8) | 7/8 (87.5%; 52.9, 97.8) |  | 36.8% | 99.0% | 8.0 (6.5-9.8) | 89.0% (94.5, 98.8) |
| Mx Neg/ND (n=21) | 18/21 (85.7%; 65.4, 95.0) |  | 60.0% | 97.0% | 7.9 (6.5-9.4) | 88.5% (82.0, 92.9) |
| BCG vacc. (n=10) | 8/10 (80.0% (49.0, 94.3) |  | 40.0% | 98.0% | 7.3 (5.9-9.2) | 88.3% (81.4, 92.9) |
| BCG uk/no vacc. (n=19) | 17/19 (89.5%; 68.6, 97.1) |  | 58.6% | 98.0% | 8.2 (6.9-9.8) | 89.1% (82.6, 93.4) |
| S (+), C (+), n=4 | 3/4 (75.0%; 30.1, 95.4) |  | 20.0% | 99.0% | 6.9 (4.7-10.1) | 88.6% (81.5, 93.2) |
| S (-), C (+), n=25 | 22/25 (88.0%; 70.0, 95.8) |  | 64.7% | 97.0% | 8.1 (6.8-9.6) | 88.9% (82.5, 93.1) |
| **Esat 6 Ag** | | | | | | |
| *EPTB cases (n=29)* | *21/29 (72.4%;54.3, 85.3)* | *73/110 (66.4%;57.1, 74.51)* | *36.2%* | *90.1%* | *2.1(2.0-2.3)* | *67.6% (59.5, 74.8)* |
| HIV Pos (n=5) | 3/5 (60.0%; 23.1, 88.2) | 73/110 (66.4%;57.1, 74.51) | 7.5% | 97.3% | 1.8 (1.1-2.9) | 66.1% (57.0, 74.1) |
| HIV Neg (n=24) | 18/24 (75.0%; 55.1, 88.0) |  | 32.7% | 92.4% | 2.2 (2.0-2.4) | 67.9% (59.6, 75.2) |
| Mx pos (n=8) | 5/8 (62.5%; 30.6, 86.3) |  | 11.9% | 96.0% | 1.9 (1.4-2.5) | 66.1% (57.2, 74.0) |
| Mx Neg/ND (n=21) | 16/21 (76.2%; 54.9, 89.4) |  | 30.2% | 93.6% | 2.3 (2.1-2.5) | 67.9% (59.5, 75.3) |
| BCG vacc. (n=10) | 7/10 (70.0%; 39.7, 89.2) |  | 15.9% | 96.0% | 2.1 (1.7-2.5) | 66.7% (57.8, 74.5) |
| BCG uk/no vacc. (n=19) | 14/19 (73.7%; 51.2, 88.2) |  | 27.4% | 93.6% | 2.2 (2.0-2.4) | 67.4% (59.0, 74.9) |
| S (+), C (+), n=4 | 4/4 (100%; 51.0, 100) |  | 9.8% | 100% | 3.0 (2.8-3.1) | 67.5% (58.5-75.4) |
| S (-), C (+), n=25 | 17/25 (68.0%; 48.4, 82.8) |  | 31.5% | 90.1% | 2.0 (1.8-2.2) | 66.7% (58.3, 74.1) |
| **Contd… Supplementary Table 2** | | | | | | |
| **Antigens/group** | **Sensitivity [n (%; 95% CI)]** | **Specificity [n (%; 95% CI)]** | **PPV (%)** | **NPV (%)** | **LRP (95% CI)** | **DA %, (95%CI)** |
| **38kDa Ag** | | | | | | |
| *EPTB cases (n=29)* | *25/29 (86.2%; 69.4, 94.5)* | *60/110 (54.5%;45.2, 63.5)* | *29.4%* | *92.6%* | *1.6 (1.5-1.6)* | *53.7% (45.7, 62.0)* |
| HIV Pos (n=5) | 4/5 (80.0%; 37.5, 96.4) | 60/110 (54.5%;45.2, 63.5) | 7.4% | 98.4% | 1.8 (1.5-2.1) | 55.6% (46.5, 64.4) |
| HIV Neg (n=24) | 21/24 (87.5%; 69.0, 95.7) |  | 29.6% | 95.2% | 1.9 (1.8-2.0) | 60.4% (52.0, 68.3) |
| Mx pos (n=8) | 7/8 (87.5%; 52.9, 97.8) |  | 12.3% | 98.4% | 1.9 (1.8-2.1) | 56.8% (47.8, 65.4) |
| Mx Neg/ND (n=21) | 19/21 (90.5%; 71.1, 97.3) |  | 27.5% | 96.8% | 2.0 (1.9-2.1) | 60.3% (51.7, 68.3) |
| BCG vacc. (n=10) | 6/10 (60.0%; 31.3, 83.2) |  | 10.7% | 93.7% | 1.3 (1.0-1.7) | 55.0% (46.1, 63.6) |
| BCG uk/no vacc. (n=19) | 19/19 (100%; 83.2, 100) |  | 27.5% | 100% | 2.2 (2.1-2.3) | 61.2% (52.6, 69.2) |
| S (+), C (+), n=4 | 4/4 (100%; 51.0, 100) |  | 7.4% | 100% | 2.2 (2.1-2.3) | 56.1% (47.0-64.9) |
| S (-), C (+), n=25 | 21/25 (84%; 65.3, 93.6) |  | 29.6% | 93.7% | 1.8 (2.7-2.0) | 60.0% (51.6, 67.9) |
| Pos: Positive, Neg: Negative, CI: Confidence interval, PPV: Positive predictive value, NPV: Negative predictive value, LRP: likelihood ratio for positive test, DA: Diagnostic accuracy, uk-unknown, Mx: Mantoux test, ND: not done, Vacc:-vaccination. C: MGIT 960 culture, S: ZN stained smear, *MDR-TB cases are DST confirmed 46 from PTB and 4 from EPTB group. | | | | | | |

**Supplementary Table 3: Comparison of dot-blot sensitivity and specificity of *M. tuberculosis*** recombinant antigens in bacteriologically confirmed MDR-TB cases (n=50; 46 PTB and 4 EPTB)

| **Antigens/group** | **Sensitivity [n (%; 95% CI)]** | **Specificity [n (%; 95% CI)]** | **PPV (%)** | **NPV (%)** | **LRP (95% CI)** | **DA %, (95%CI)** |
| --- | --- | --- | --- | --- | --- | --- |
| **Rv2970c (lipN)** | | | | | | |
| *MDR-TB (n=50)* | *50/50 (100%; 92.9, 100)* | *108/110 (98.2%;93.6,99.6)* | *96.1%* | *100%* | *55.0 (20.6-146.5)* | *98.7% (95.6, 99.7)* |
| HIV Neg (n=50) | 50/50 (100%; 89.8, 100) | 108/110 (98.2%;93.6,99.6) | 96.1% | 100% | 55.0 (20.6-146.5) | 98.7% (95.6, 99.7) |
| Mx pos (n=34) | 34/34 (100%; 89.8, 100) |  | 94.4% | 100% | 55.0 (20.6-146.5) | 98.6% (95.1, 99.6) |
| Mx Neg/ND (n=16) | 16/16 (100% (80.6, 100) |  | 88.9% | 100% | 55.0 (20.6-146.5) | 98.4% (94.4, 99.6) |
| BCG vacc. (n=20) | 20/20 (100%; 83.9, 100) |  | 90.9% | 100% | 55.0 (20.6-146.5) | 98.5% (94.6, 99.6) |
| BCG uk/no vacc. (n=30) | 30/30 (100%; 88.6, 100) |  | 93.5% | 100% | 55.0 (20.6-146.5) | 98.6% (94.9, 99.6) |
| S (+), C (+), n=50 | 50/50 (100%; 92.9, 100) |  | 96.1% | 100% | 55.0 (20.6-146.5) | 98.7% (95.6, 99.7) |
| **Rv2145c (wag31)** | | | | | | |
| *MDR-TB (n=50)* | *50/50 (100%; 92.9, 100)* | *110/110 (100%;96.6,100)* | *100%* | *100%* | *-* | *100% (97.7, 100)* |
| HIV Neg (n=50) | 50/50 (100%; 92.9, 100) | 110/110 (100%;96.6,100) | 100% | 100% | - | 100% (97.7, 100) |
| Mx pos (n=34) | 34/34 (100%; 89.8, 100) |  | 100% | 100% | - | 100% (97.4, 100) |
| Mx Neg/ND (n=16) | 16/16 (100%; 80.6, 100) |  | 100% | 100% | - | 100% (94.0, 100) |
| BCG vacc. (n=20) | 20/20 (100%; 83.9, 100) |  | 100% | 100% | - | 100% (97.1, 100) |
| BCG uk/no vacc. (n=30) | 30/30 (100%; 88.6, 100) |  | 100% | 100% | - | 100% (97.3, 100) |
| S (+), C (+), n=50 | 50/50 (100%; 92.9, 100) |  | 100% | 100% | - | 100% (97.7, 100) |
| **Rv1827 (garA)** | | | | | | |
| *MDR-TB (n=50)* | *50/50 (100%; 92.9, 100)* | *103/110 (93.6%;87.4, 96.9)* | *87.7%* | *100%* | *15.7 (11.9-20.8)* | *95.6% (91.2, 97.9)* |
| HIV Neg (n=50) | 50/50 (100%; 92.9, 100) | 103/110 (93.6%;87.4, 96.9) | 87.7% | 100% | 15.7 (11.9-20.8) | 95.6% (91.2, 97.9) |
| Mx pos (n=34) | 34/34 (100%; 89.8, 100) |  | 82.9% | 100% | 15.7 (11.9-20.8) | 95.1% (90.3, 97.6) |
| Mx Neg/ND (n=16) | 16/16 (100%; 80.6, 100) |  | 69.6% | 100% | 15.7 (11.9-20.8) | 94.4% (89.0, 97.3) |
| BCG vacc. (n=20) | 20/20 (100%; 83.9, 100) |  | 74.1% | 100% | 15.7 (11.9-20.8) | 94.6% (89.3, 97.4) |
| BCG uk/no vacc. (n=30) | 30/30 (100%; 88.6, 100) |  | 81.1% | 100% | 15.7 (11.9-20.8) | 95.0% (90.0, 97.6) |
| S (+), C (+), n=50 | 24/24 (100%; 86.2, 100) |  | 77.4% | 100% | 15.7 (11.9-20.8) | 94.8% (89.6, 97.4) |

| **Contd… Supplementary Table 3** | | | | | | |
| --- | --- | --- | --- | --- | --- | --- |
| **Antigens/group** | **Sensitivity [n (%; 95% CI)]** | **Specificity [n (%; 95% CI)]** | **PPV (%)** | **NPV (%)** | **LRP (95% CI)** | **DA %, (95%CI)** |
| **Rv0164 (TB 18.5)** | | | | | | |
| *MDR-TB (n=50)* | *50/50 (100%; 92.9, 100)* | *102/110 (92.7%;86.3, 96.3)* | *86.2%* | *100%* | *13.7 (10.8-17.6)* | *95.0% (90.4, 97.4)* |
| HIV Neg (n=50) | 50/50 (100%; 92.9, 100 | 102/110 (92.7%;86.3, 96.3) | 86.2% | 100% | 13.7 (10.8-17.6) | 95.0% (90.4, 97.4) |
| Mx pos (n=34) | 34/34 (100%; 89.8, 100) |  | 80.9% | 100% | 13.7 (10.8-17.6) | 94.4% (89.4, 97.2) |
| Mx Neg/ND (n=16) | 16/16 (100%; 80.6, 100) |  | 69.6% | 100% | 13.7 (10.8-17.6) | 93.6% (88.0, 96.7) |
| BCG vacc. (n=20) | 20/20 (100%; 83.9, 100) |  | 71.4% | 100% | 13.7 (10.8-17.6) | 93.8% (88.3, 96.8) |
| BCG uk/no vacc. (n=30) | 30/30 (100%; 88.6, 100) |  | 78.9% | 100% | 13.7 (10.8-17.6) | 94.3% (89.1, 97.1) |
| S (+), C (+), n=50 | 24/24 (100%; 86.2, 100) |  | 75.0% | 100% | 13.7 (10.8-17.6) | 94.0% (88.7, 96.9) |
| **Rv1437 (pgk)** | | | | | | |
| *MDR-TB (n=50)* | *50/50 (100%; 92.9, 100)* | *98/110 (89.1%;81.9, 93.6)* | *80.6%* | *100%* | *9.2 (7.8-10.8)* | *92.5% (87.3, 95.7)* |
| HIV Neg (n=50) | 50/50 (100%; 92.9, 100) | 98/110 (89.1%;81.9, 93.6) | 80.6% | 100% | 9.2 (7.8-10.8) | 92.5% (87.3, 95.7) |
| Mx pos (n=34) | 34/34 (100%; 89.8, 100) |  | 73.9% | 100% | 9.2 (7.8-10.8) | 91.7% (86.0, 95.2) |
| Mx Neg/ND (n=16) | 16/16 (100%; 80.6, 100) |  | 57.1% | 100% | 9.2 (7.8-10.8) | 90.5% (84.1, 94.5) |
| BCG vacc. (n=20) | 20/20 (100%; 83.9, 100) |  | 62.5% | 100% | 9.2 (7.8-10.8) | 90.8% (84.6, 94.6) |
| BCG uk/no vacc. (n=30) | 30/30 (100%; 88.6, 100) |  | 71.4% | 100% | 9.2 (7.8-10.8) | 91.4% (85.6, 95.0) |
| S (+), C (+), n=50 | 24/24 (100%; 86.2, 100) |  | 66.7% | 100% | 9.2 (7.8-10.8) | 91.0% (85.0, 94.8) |
| **Esat 6 Ag** | | | | | | |
| *MDR-TB (n=50)* | *45/50 (90.0%;78.6, 95.6 )* | *73/110 (66.4%;57.1, 74.51)* | *54.9%* | *93.6%* | *2.7 (2.5 - 2.8)* | *73.7%(66.4, 79.9)* |
| HIV Neg (n=50) | 45/50 (90.0%;78.6, 95.6 ) | 73/110 (66.4%;57.1, 74.51) | 54.9% | 93.6% | 2.7 (2.5 - 2.8) | 73.7%(66.4, 79.9) |
| Mx pos (n=34) | 31/34 (91.2%; 77.0, 96.9) |  | 45.6% | 96.1% | 2.7 (2.6-2.9) | 72.2% (64.4, 78.9) |
| Mx Neg/ND (n=16) | 14/16 (87.5%; 64.0, 96.5) |  | 27.5% | 97.3% | 2.6 (2.4-2.8) | 69.0% (60.5, 76.5) |
| BCG vacc. (n=20) | 17/20 (85.0%; 64.0, 94.8) |  | 31.5% | 96.0% | 2.5 (2.3-2.7( | 69.2% (60.8, 76.5) |
| BCG uk/no vacc. (n=30) | 28/30 (93.3%; 78.7, 98.1) |  | 43.1% | 97.3% | 2.8 (2.6-2.9) | 72.1% (64.2, 78.9) |
| S (+), C (+), n=50 | 45/50 (90.0%;78.6, 95.6 ) |  | 54.9% | 93.6% | 2.7 (2.5 - 2.8) | 73.7%(66.4, 79.9) |
| **38kDa Antigen** | | | | | | |
| *MDR-TB (n=50)* | *47/50 (94.0%;83.8, 97.9)* | *60/110 (54.5%;45.2, 63.5)* | *43.9%* | *94.3%* | *1.7 (1.7-1.8)* | *60.6% (52.9, 67.9)* |
| HIV Neg (n=50) | 50/50 (100%; 92.9, 100) | 60/110 (54.5%;45.2, 63.5) | 50.0% | 100% | 2.2 (2.1-2.3) | 68.7% (61.2, 75.4) |
| Mx pos (n=34) | 31/34 (91.2%; 77.0, 96.9) |  | 38.3% | 95.2% | 2.0 (1.9-2.1) | 63.2% (55.1, 70.6) |
| Mx Neg/ND (n=16) | 16/16 (100%; 80.6, 100) |  | 24.2% | 100% | 2.2 (2.1-2.3) | 60.3% (51.6, 68.4) |
| BCG vacc. (n=20) | 18/20 (90.0%; 69.9, 97.2) |  | 26.5% | 96.8% | 2.0 (1.9-2.1) | 60.0% (51.4, 68.0) |
| BCG uk/no vacc. (n=30) | 29/30 (96.7%; 83.3, 99.4) |  | 36.7% | 98.4% | 2.1 (2.0-2.2) | 63.6% (55.3, 71.1) |
| S (+), C (+), n=50 | 50/50 (100%; 92.9, 100) |  | 50.0% | 100% | 2.2 (2.1-2.3) | 68.7% (61.2, 75.4) |
| Pos: Positive, Neg: Negative, CI: Confidence interval, PPV: Positive predictive value, NPV: Negative predictive value, LRP: likelihood ratio for positive test, DA: Diagnostic accuracy, uk-unknown, Mx: Mantoux test, ND: not done, Vacc:-vaccination. C: MGIT 960 culture, S: ZN stained smear, *MDR-TB cases are DST confirmed 46 from PTB and 4 from EPTB group | | | | | | |

**Supplementary Table 4: Comparison of ELISA sensitivity and specificity of *M. tuberculosis*** antigens in bacteriologically confirmed PTB cases (n=111)

| **Antigens/group** | **Sensitivity [n (%; 95% CI)]** | **Specificity [n (%; 95% CI)]** | **PPV (%)** | **NPV (%)** | **LRP (95% CI)** | **DA %, (95%CI)** |
| --- | --- | --- | --- | --- | --- | --- |
| **Rv2970c (lipN)** | | | | | | |
| *PTB cases (n=111)* | *107/111(96.4%; 91.1, 98.6)* | *108/110 (98.2%;93.6,99.5)* | *98.2%* | *96.4%* | *53.2(19.9-141.4)* | *97.3% (94.2, 98.7)* |
| HIV Pos (n=15) | 13/15 (86.67%; 62.1, 96.3) | 108/110 (98.2%;93.6,99.5) | 86.7% | 98.2% | 47.7 (17.5-130) | 96.8% (92.1, 98.7) |
| HIV Neg (n=96) | 94/96 (97.9%; 92.7, 99.4) |  | 97.9% | 98.2% | 53.8 (20.2-143.6) | 98.1% (95.1, 99.2) |
| Mx pos (n=68) | 66/68 (97.1%; 89.9, 99.2) |  | 97.1% | 98.2% | 53.4 (20.0-142.4) | 97.7% (94.4, 99.1) |
| Mx Neg/ND (n=43) | 41/43 (95.3%; 84.5, 98.7) |  | 95.3% | 98.2% | 52.4 (19.6-140.1) | 97.4% (93.5, 99.0) |
| BCG vacc. (n=42) | 39/42 (92.9%; 81.0, 97.5) |  | 95.1% | 97.3% | 51.1 (19.1-136.6) | 96.7% (92.5, 98.6) |
| BCG uk/no vacc. (n=69) | 68/69 (98.5%; 92.2, 99.7) |  | 97.1% | 99.1% | 54.2 (20.3-144.5) | 98.3% (95.2, 99.4) |
| S (+), C (+), n=58 | 58/58 (100%; 93.8, 100) |  | 96.7% | 100% | 55.0 (20.6-146.5) | 98.8% (95.8, 99.7) |
| S (-), C (+), n=53 | 49/53 (92.4%; 82.1, 97.0) |  | 96.1% | 96.4% | 50.8 (19.0-135.9) | 96.3% (92.2, 98.3) |
| **Rv2145c (wag31)** | | | | | | |
| *PTB cases (n=111)* | *107/111 (96.4%; 91.1,98.6)* | *107/110 (97.3%;92.3,99.1)* | *97.3%* | *96.4%* | *35.3 (18.4-68.0)* | *96.8% (93.6, 98.5)* |
| HIV Pos (n=15) | 13/15 (86.7%; 62.1, 96.3) | 107/110 (97.3%;92.3,99.1) | 81.2% | 98.2% | 31.8 (16.2-62.5) | 96.0% (91.0, 98.3) |
| HIV Neg (n=96) | 94/96 (97.9%; 92.7,99.4) |  | 96.9% | 98.2% | 35.9 (18.7-69.0) | 97.6% (94.4, 99.0) |
| Mx pos (n=68) | 68/68 (100%; 96.2, 100) |  | 95.8% | 100% | 37.7 (19.1-99.4) | 98.3% (95.2, 99.4) |
| Mx Neg/ND (n=43) | 37/43 (86.0%; 72.7, 93.4) |  | 92.5% | 94.7% | 31.5 (16.3-61.2) | 94.1% (89.2, 97.0) |
| BCG vacc. (n=42) | 41/42 (97.6%; 87.7, 99.6) |  | 93.2% | 99.1% | 35.8 (18.6-68.9) | 97.4% (93.4, 99.0) |
| BCG uk/no vacc.(n=69) | 66/69 (95.6%; 88.0,98.5) |  | 95.6% | 97.3% | 35.1 (18.2-67.5) | 96.6% (92.9, 98.4) |
| S (+), C (+), n=58 | 58/58 (100%; 93.8, 100) |  | 95.1% | 100% | 36.7 (19.1-70.5) | 98.2% (94.9, 99.4) |
| S (-), C (+), n=53 | 49/53 (92.4%; 82.1, 97.0) |  | 94.2% | 96.4% | 33.9 (17.6-65.4) | 95.7% (91.4, 97.9) |
| **Rv1827 (garA)** | | | | | | |
| *PTB cases (n=111)* | *102/111(91.9%; 85.3, 95.7)* | *104/110 (94.5%;88.6, 97.5)* | *94.5%* | *92.0%* | *16.8 (12.1-23.4)* | *93.2% (89.1, 95.8)* |
| HIV Pos (n=15) | 13/15 (86.7%; 62.1, 96.3) | 104/110 (94.5%;88.6, 97.5) | 68.4% | 98.1% | 15.9 (11.2-22.5) | 93.6% (87.9, 96.7) |
| HIV Neg (n=96) | 89/96 (92.7%; 85.7, 96.4) |  | 93.7% | 93.7% | 17.0 (12.2-23.6) | 93.7% (89.5, 96.3) |
| Mx pos (n=68) | 63/68 (92.6%; 83.9, 96.8) |  | 91.3% | 95.4% | 17.0 (12.2-23.6) | 93.8% (89.3, 96.5) |
| Mx Neg/ND (n=43) | 39/43 (90.7%; 78.4, 96.3) |  | 86.7% | 96.3% | 16.6 (11.9-23.2) | 93.5% (88.4, 96.4) |
| BCG vacc. (n=42) | 41/42 (97.6%; 87.7, 99.6) |  | 87.2% | 99.0% | 17.9 (12.9-24.8) | 95.4% (90.8, 97.7) |
| BCG uk/no vacc. (n=69) | 61/69 (88.4%; 78.7, 94.0) |  | 91.0% | 92.9% | 16.2 (11.6-22.6) | 92.2% (87.3, 95.3) |
| S (+), C (+), n=58 | 55/58 (94.83%; 85.9, 98.2) |  | 90.2% | 97.2% | 17.4 (12.5-24.1) | 94.6% (90.1, 97.2) |
| S (-), C (+), n=53 | 47/58 (81.0%; 69.1, 89.1) |  | 88.7% | 90.4% | 14.9 (10.6-20.8) | 89.9% (84.4, 93.6) |
| **Contd… Supplementary Table 4** | | | | | | |
| **Antigens/group** | **Sensitivity [n (%; 95% CI)]** | **Specificity [n (%; 95% CI)]** | **PPV (%)** | **NPV (%)** | **LRP (95% CI)** | **DA %, (95%CI)** |
| **Rv0164 (TB 18.5)** | | | | | | |
| *PTB cases (n=111)* | *81/111 (73.0%; 64.0,80.4)* | *96/110 (87.3%;79.8,92.3)* | *85.3%* | *76.2%* | *5.7(4.9–6.6)* | *80.1% (74.3, 84.8)* |
| HIV Pos (n=15) | 8/15 (53.3%; 30.1, 75.2) | 96/110 (87.3%;79.8,92.3) | 36.4% | 93.2% | 4.2 (2.9-6.0) | 83.2% (75.7, 88.7) |
| HIV Neg (n=96) | 73/96 (76.0%; 66.6, 83.5) |  | 83.9% | 80.7% | 6.0 (5.1-6.9) | 82.0% (76.2, 86.7) |
| Mx pos (n=68) | 48/68 (70.6%; 58.9, 80.1) |  | 77.4% | 82.8% | 5.5 (4.7-6.5) | 80.9% (74.5, 86.0) |
| Mx Neg/ND (n=43) | 33/43 (76.7%; 62.3, 86.8) |  | 70.2% | 90.6% | 6.0 (5.1-7.1) | 84.3% (77.7, 89.2) |
| BCG vacc. (n=42) | 30/42 (71.4%; 56.4, 82.8) |  | 68.2% | 99.9% | 5.6 (4.7-6.6) | 82.9% (76.1, 88.0) |
| BCG uk/no vacc.(n=69) | 51/69 (73.9%; 62.5, 82.8) |  | 78.5% | 84.2% | 5.8 (5.0-6.8) | 82.1% (75.8, 87.0) |
| S (+), C (+), n=58 | 41/58 (70.7%; 58.0, 80.8) |  | 74.5% | 85.0% | 5.5 (4.7-6.5) | 81.5% (75.0, 86.7) |
| S (-), C (+), n=53 | 43/53 (81.13%; 68.6, 89.4) |  | 75.4% | 90.6% | 6.4 (5.5-7.4) | 85.3% (790, 89.9) |
| **Rv1437 (pgk)** | | | | | | |
| *PTB cases (n=111)* | *82/111 (73.9%; 65.0, 81.1)* | *95/110 (86.7%;78.7, 91.6)* | *84.5%* | *76.6%* | *5.4 (4.7-6.2)* | *80.1% (74.3, 84.8)* |
| HIV Pos (n=15) | 8/15 (53.3%; 30.1, 75.2) | 95/110 (86.7%;78.7, 91.6) | 34.8% | 93.1% | 3.9 (2.8-5.5) | 82.4% (74.8, 88.1) |
| HIV Neg (n=96) | 74/96 (77.1%; 67.7, 84.3) |  | 83.1% | 81.2% | 5.6 (4.9-6.5) | 82.0% (76.2, 86.7) |
| Mx pos (n=68) | 52/68 (76.5%; 65.1, 85.0) |  | 77.6% | 85.6% | 5.6 (4.7-6.5) | 82.6% (76.3, 87.4) |
| Mx Neg/ND (n=43) | 30/43 (69.8%; 55.0, 81.4) |  | 66.7% | 88.0% | 5.1 (4.3-6.0) | 81.7% (74.8, 87.0) |
| BCG vacc. (n=42) | 30/42 (71.4%; 56.4, 82.8) |  | 66.7% | 88.8% | 5.2 (4.5-6.1) | 82.2% (75.4, 87.5) |
| BCG uk/no vacc. (n=69) | 52/69 (82.2%; 75.4, 87.5) |  | 77.6% | 84.8% | 5.5 (4.8-6.4) | 82.1% (75.8, 87.0) |
| S (+), C (+), n=58 | 44/58 (75.7%; 63.5, 85.0) |  | 74.6% | 87.2% | 5.6 (4.8-6.4) | 82.7% (76.3, 87.7) |
| S (-), C (+), n=53 | 40/53 (75.5%; 62.4, 85.1) |  | 72.7% | 88.0% | 5.5 (4.8-6.4) | 82.8% (76.3, 87.8) |
| **Esat6 Ag** | | | | | | |
| *PTB cases (n=111)* | *81/111 (72.97%;64.0, 80.4)* | *78/110 (70.9%;61.8, 78.6)* | *71.7%* | *72.2* | *2.5 (2.3-2.7)* | *71.9% (65.7-77.5)* |
| HIV Pos (n=15) | 11/15 (73.3%; 48.0, 89.1) | 78/110 (70.9%;61.8, 78.6) | 25.6% | 95.1% | 2.5 (2.2-2.9) | 71.2% (62.7, 78.4) |
| HIV Neg (n=96) | 84/96 (87.5%; 79.4, 92.7) |  | 72.4% | 86.7% | 3.0 (2.8-3.2) | 78.6% (72.5, 83.7) |
| Mx pos (n=68) | 62/68 (91.2%; 82.1, 95.9) |  | 66.0% | 92.9% | 3.1 (2.9-3.3) | 78.6% (72.1, 84.0) |
| Mx Neg/ND (n=43) | 33/43 (76.7%; 62.3, 86.8) |  | 50.8% | 88.6% | 2.6 (24-1.9) | 72.5% (65.0, 79.0) |
| BCG vacc. (n=42) | 35/42 (83.33%; 69.4, 91.7) |  | 52.2% | 91.8% | 2.9 (2.7-3.1) | 74.3% (66.9, 80.6) |
| BCG uk/no vacc. (n=69) | 60/69 (87.0%; 77.0, 93.0) |  | 65.2% | 89.7% | 3.0 (2.8-3.2 | 77.1% (70.4, 82.6) |
| S (+), C (+), n=58 | 38/58 (65.5%; 52.7, 76.4) |  | 54.3% | 79.6% | 2.2 (2.1-2.5) | 69.0% (61.7, 75.5) |
| S (-), C (+), n=53 | 43/53 (81.1%; 68.6, 89.4) |  | 57.3% | 88.6% | 2.8 (2.6-3.0) | 74.2% (67.0, 80.3) |
| **Contd… Supplementary Table 4** | | | | | | |
| **Antigens/group** | **Sensitivity [n (%; 95% CI)]** | **Specificity [n (%; 95% CI)]** | **PPV (%)** | **NPV (%)** | **LRP (95% CI)** | **DA %, (95%CI)** |
| **38kDa Ag** |  |  |  |  |  |  |
| *PTB cases (n=111)* | *103/11 (92.8%;86.4, 96.3)* | *67/110 (60.9%;51.6, 69.5)* | *70.5%* | *89.3%* | *2.4 (2.3-2.5)* | *76.9% (70.9, 82.0)* |
| HIV Pos (n=15) | 11/15 (73.3%; 48.0, 89.1) | 67/110 (60.9%;51.6, 69.5) | 20.4% | 94.4% | 1.9 (1.7-2.1) | 62.4% (53.7, 70.4) |
| HIV Neg (n=96) | 84/96 (87.5%; 79.4, 92.7) |  | 66.1% | 84.8% | 2.2 (2.1-2.3) | 73.3% (66.9, 78.9) |
| Mx pos (n=68) | 60/68 (88.2%; 78.5, 93.9) |  | 58.2% | 89.3% | 2.3 (2.1-2.4) | 71.3% (64.3, 77.5) |
| Mx Neg/ND (n=43) | 35/43 (81.4%; 67.4, 90.3) |  | 44.9% | 89.3% | 2.1 (2.0-2.2) | 66.7% (58.9, 73.6) |
| BCG vacc. (n=42) | 36/42 (88.1%; 75, 9.81.0) |  | 46.2% | 93.1% | 2.2 (2.1-2.4) | 68.4% (60.6, 75.3) |
| BCG uk/no vacc. (n=69) | 59/69 (85.5%; 75.3, 91.9) |  | 57.8% | 87.0% | 2.2 (2.1-2.3) | 70.4% (63.3, 76.6) |
| S (+), C (+), n=58 | 54/58 (93.1%; 83.6, 97.3) |  | 55.7% | 94.4% | 2.4 (2.3-2.5) | 72.0% (64.8, 78.3) |
| S (-), C (+), n=53 | 49/53 (92.4%; 82.1, 97.0) |  | 53.3% | 94.4% | 2.4 (2.2-2.5) | 71.2% (63.8, 77.6) |
| Pos: Positive, Neg: Negative, CI: Confidence interval, PPV: Positive predictive value, NPV: Negative predictive value, LRP: likelihood ratio for positive test, DA: Diagnostic accuracy, uk: unknown, Mx: Mantoux test, ND: not done, Vacc.-Vaccination. C: MGIT 960 culture, S: ZN stained smear, *MDR-TB cases are DST confirmed 46 from PTB and 4 from EPTB group, P value <0.001 (control vs patient), P_Rv2970c_ =<0.001, P_Rv2145c_ =0.008 and P_Rv1827_=<0.001 (sensitive vs MDR-TB). | | | | | | |

**Supplementary Table 5: Comparison of ELISA sensitivity and specificity of *M. tuberculosis* antigens in bacteriologically confirmed EPTB cases (n=29)**

| **Antigens/group** | **Sensitivity [n (%; 95% CI)]** | **Specificity [n (%; 95% CI)]** | **PPV (%)** | **NPV (%)** | **LRP (95% CI)** | **DA %, (95%CI)** |
| --- | --- | --- | --- | --- | --- | --- |
| **Rv2970c (lipN)** | | | | | | |
| *EPTB cases (n=29)* | *28/29(96.5%; 82.8, 99.4)* | *108/110 (98.2%;93.6,99.5)* | *93.3%* | *99.1%* | *53.1 (19.9-141.8)* | *97.8% (93.8, 99.3)* |
| HIV Pos (n=5) | 5/5 (100%; 56.5, 100) | 108/110 (98.2%;93.6,99.5) | 71.4% | 100% | 55 (20.6-146.5) | 98.3% (93.9, 99.5) |
| HIV Neg (n=24) | 23/24 (95.8%; 79.8, 99.3) |  | 92.0% | 99.1% | 52.7 (19.7-141) | 97.8% (93.6, 99.2) |
| Mx pos (n=8) | 8/8 (100%; 67.6, 100) |  | 80.0% | 100% | 55.0 (20.6-146.5) | 98.3% (94.0, 99.5) |
| Mx Neg/ND (n=21) | 20/21 (95.2%; 77.3, 99.1) |  | 90.1% | 99.1% | 52.4 (19.6-140.3) | 97.7% (93.5, 99.2) |
| BCG vacc. (n=10) | 10/10 (100%; 72.2, 100) |  | 83.3% | 100% | 55 (20.6-146.5) | 98.3% (94.1, 99.5) |
| BCG uk/no vacc. (n=19) | 18/19 (94.7%; 75.4, 99.1) |  | 90.0% | 99.1% | 52.1 (19.4-139.7) | 97.7% (93.4, 99.2) |
| S (+), C (+), n=4 | 4/4 (100%; 51.0, 100) |  | 66.6% | 100% | 55 (20.6-146.5) | 98.2% (93.8,99.5) |
| S (-), C (+), n=25 | 24/25 (96.0%; 80.5, 99.3) |  | 92.6% | 99.1% | 52.8 (19.7-141.2) | 97.8% (93.7, 99.2) |
| **Rv2145c (wag31)** | | | | | | |
| *EPTB cases (n=29)* | *26/29 (89.7%; 73.6,96.4)* | *107/110 (97.3%;92.3,99.1)* | *89.7%* | *97.3%* | *32.9 (17.0-63.7)* | *95.7% (90.9, 98.0)* |
| HIV Pos (n=5) | 4/5 (80.0%; 37.5, 96.4) | 107/110 (97.3%;92.3,99.1) | 57.1% | 99.1% | 29.3 (13.5-63.7) | 96.5% (91.4, 98.6) |
| HIV Neg (n=24) | 22/24 (91.7%; 74.1, 97.7) |  | 88.0% | 98.2% | 33.6 (17.3-65.1) | 96.3% (91.6, 98.4) |
| Mx pos (n=8) | 8/8 (100%; 97.6, 100) |  | 72.7% | 100% | 36.7 (19.1-70.5) | 97.5% (92.8, 99.1) |
| Mx Neg/ND (n=21) | 18/21 (85.7%; 65.4, 95.0) |  | 85.7% | 97.3% | 31.4 (16.1-61.5) | 95.4% (90.4, 97.9) |
| BCG vacc. (n=10) | 8/10 (80.0%; 49.0, 94.3) |  | 72.7% | 98.2% | 29.3 (14.4-59.9) | 95.8% (90.6, 98.2) |
| BCG uk/no vacc.(n=19) | 18/19 (94.7%; 75.4, 99.1) |  | 85.7% | 99.1% | 34.7 (18.0-67.2) | 96.9% (92.4, 98.8) |
| S (+), C (+), n=4 | 4/4 (100%; 51.0, 100) |  | 57.1% | 100% | 36.7 (19.1-70-5) | 97.4% (92.5, 99.1) |
| S (-), C (+), n=25 | 22/25 (88.0%; 70.0, 95.8) |  | 88.0% | 97.3% | 32.3 (16.6-62.8) | 95.6% (90.6, 97.9) |
| **Rv1827 (garA)** | | | | | | |
| *EPTB cases (n=29)* | *26/29(89.7%; 73.6, 96.4)* | *104/110(94.5%;88.6, 97.5)* | *81.2%* | *97.2%* | *16.4 (11.7-23.0)* | *93.5% (88.1, 96.6)* |
| HIV Pos (n=5) | 5/5 (100%; 56.5, 100) | 104/110(94.5%;88.6, 97.5) | 45.4% | 100% | 18.3 (13.2-25.4) | 94.8% (89.1, 97.6) |
| HIV Neg (n=24) | 21/24 (87.5%; 69.0, 95.7) |  | 77.8% | 97.2% | 16.0 (11.4-22.5) | 93.3% (87.7, 96.4) |
| Mx pos (n=8) | 8/8 (100%; 67.6, 100) |  | 57.1% | 100% | 18.3 (13.2-25.4) | 94.9% (89.3, 97.6) |
| Mx Neg/ND (n=21) | 18/21 (85.7%; 65.4, 95.0) |  | 75.0% | 97.2% | 15.7 (11.1-22.2) | 93.1% (87.5, 96.3) |
| BCG vacc. (n=10) | 9/10 (90.0% (59.6, 98.2) |  | 60.0% | 99.0% | 16.5 (11.6-23.4) | 94.2%(88.4, 97.1) |
| BCG uk/no vacc. (n=19) | 16/19 (84.2%; 62.4, 94.5) |  | 72.7% | 97.2% | 15.4 (10.9-21.9) | 93.0% (87.3, 96.3) |
| S (+), C (+), n=4 | 4/4 (100%; 51.0, 100) |  | 40.0% | 100% | 18.3 (13.2-25.4) | 94.7% (89.0, 97.6) |
| S (-), C (+), n=25 | 24/25 (96.0%; 80.5, 99.3) |  | 80.0% | 99.0% | 17.6 (12.6-24.5) | 94.8% (89.7, 97.5) |

| **Contd… Supplementary Table 5** | | | | | | |
| --- | --- | --- | --- | --- | --- | --- |
| **Antigens/group** | **Sensitivity [n (%; 95% CI)]** | **Specificity [n (%; 95% CI)]** | **PPV (%)** | **NPV (%)** | **LRP (95% CI)** | **DA %, (95%CI)** |
| **Rv0164 (TB 18.5)** | | | | | | |
| *EPTB cases (n=29)* | *20/29(69.0%; 50.8, 82.7)* | *96/110 (87.3%;79.8,92.3)* | *58.8%* | *91.4%* | *5.4 (4.5-6.5)* | *83.4% (76.4, 88.7)* |
| HIV Pos (n=5) | 3/5 (60.0%; 23.1, 88.2) | *96/110 (87.3%;79.8,92.3)* | 17.6% | 98.0% | 4.7 (2.6-8.4) | 86.1% (78.6, 91.2) |
| HIV Neg (n=24) | 17/24 (70.8%; 50.8, 85.1) |  | 54.8% | 93.2% | 5.6 (4.6-6.7) | 84.4% (77.2, 89.5) |
| Mx pos (n=8) | 7/8 (87.5%; 52.9, 97.8) |  | 33.4% | 99.0% | 6.9 (5.7-8.2) | 87.3% (80.1, 92.1) |
| Mx Neg/ND (n=21) | 13/21 (61.9%; 40.9, 79.2) |  | 48.1% | 92.3% | 4.9 (3.8-6.1) | 83.2% (75.9, 88.6) |
| BCG vacc. (n=10) | 6/10 (60.0%; 31.3, 83.2) |  | 30.0% | 96.0% | 4.7 (3.3-6.7) | 85.0% (77.5, 90.3) |
| BCG uk/no vacc.(n=19) | 14/19 (73.7%; 51.2, 88.2) |  | 50.0% | 95.0% | 5.8 (4.8-7.0) | 85.3% (78.1, 90.4) |
| S (+), C (+), n=4 | 3/4 (75.0%; 30.1, 95.4) |  | 17.6% | 99.0% | 5.9 (4.1-8.4) | 86.8% (79.4, 91.9) |
| S (-), C (+), n=25 | 17/25 (68.0%; 48.4, 82.8) |  | 54.8% | 92.3% | 5.3 (4.4-6.5) | 83.7% (76.6, 89.0) |
| **Rv1437 (pgk)** | | | | | | |
| *EPTB cases (n=29)* | *22/29 (75.9%; 57.9, 87.8)* | *95/110 (86.7%;78.7, 91.6)* | *59.5%* | *93.1%* | *5.6 (4.7-6.5)* | *84.2% (77.2, 89.3)* |
| HIV Pos (n=5) | 4/5 (80.0%; 37.5, 96.4) | 95/110 (86.7%;78.7, 91.6) | 21.0% | 98.7% | 5.9 (4.5-7.6) | 86.1% (78.6, 91.2) |
| HIV Neg (n=24) | 18/24 (75.0%; 55.1, 88.0) |  | 54.6% | 86.4% | 5.5 (4.6-6.5) | 84.3% (77.2, 89.5) |
| Mx pos (n=8) | 7/8 (87.5%; 52.9, 97.8) |  | 31.8% | 99.0% | 6.4 (5.4-7.6) | 86.4% (79.1, 91.4) |
| Mx Neg/ND (n=21) | 15/21 (71.4%; 50.0, 86.2) |  | 50.0% | 94.1% | 5.2 (4.3-6.3) | 84.0% (76.7, 89.3) |
| BCG vacc. (n=10) | 6/10 (60.0%; 31.3, 83.2) |  | 28.6% | 96.0% | 4.4 (3.1-6.2) | 84.2% (76.6, 89.6) |
| BCG uk/no vacc. (n=19) | 16/19 (84.2%; 62.4, 94.5) |  | 51.6% | 96.9% | 6.2 (5.3-7.2) | 86.0% (79.0, 91.0) |
| S (+), C (+), n=4 | 3/4 (75.0%; 30.1, 95.4) |  | 16.7% | 99.0% | 5.5 (3.8-7.8) | 86.0% (78.4, 91.2) |
| S (-), C (+), n=25 | 19/25 (76.0%; 56.6, 88.5) |  | 55.9% | 94.1% | 5.6 (4.7-6.6) | 84.4% (77.4, 89.6) |
| **Esat6 Ag** | | | | | | |
| *EPTB cases (n=29)* | *26/29 (89.7%; 73.6,96.4)* | *78/110 (70.9%;61.8, 78.6)* | *44.8%* | *96.3%* | *3.1 (2.9-3.3)* | *74.8% (67.8, 81.3)* |
| HIV Pos (n=5) | 3/5 (60.0%; 23.1, 88.2) | 78/110 (70.9%;61.8, 78.6) | 8.6% | 97.5% | 2.1 (1.2-3.4) | 70.4% (61.5, 78.0) |
| HIV Neg (n=24) | 18/24 (75.0%; (55.1, 88.0) |  | 36.0% | 92.9% | 2.6 (2.4-2.8) | 71.6%; 63.5, 78.6) |
| Mx pos (n=8) | 5/8 (62.5%; 30.6, 86.3) |  | 13.5% | 96.3% | 2.2 (1.6-2.9) | 70.3% (61.6, 77.8) |
| Mx Neg/ND (n=21) | 16/21 (76.2%; 54.9, 89.4) |  | 33.3% | 94.0% | 2.6 (2.4-2.9) | 71.8% (63.5, 78.8) |
| BCG vacc. (n=10) | 7/10 (70%; 39.7, 89.2) |  | 17.9% | 96.3% | 2.4 (2.0-2.9) | 70.8% (62.2, 78.2) |
| BCG uk/no vacc. (n=19) | 14/19 (73.7%; 51.2, 88.2) |  | 30.4% | 94.0% | 2.5 (2.3-1.8) | 71.3% (63.0, 78.4) |
| S (+), C (+), n=4 | 4/4 (100%; 51.0, 100) |  | 11.1% | 100% | 3.4 (3.2-3.7) | 71.9% (63.1, 79.4) |
| S (-), C (+), n=25 | 17/25 (68%; 48.4, 82.8) |  | 34.% | 90.7% | 2.4 (2.1-2.6) | 70.4% (62.2, 77.4) |
| **Contd… Supplementary Table 5** | | | | | | |
| **Antigens/group** | **Sensitivity [n (%; 95% CI)]** | **Specificity [n (%; 95% CI)]** | **PPV (%)** | **NPV (%)** | **LRP (95% CI)** | **DA %, (95%CI)** |
| **38kDa Ag** | | | | | | |
| *EPTB cases (n=29)* | *26/29 (89.7%; 73.6,96.4)* | *67/110 (60.9%;51.6, 69.5)* | *37.7%* | *95.7%* | *2.3 (2.2-2.4)* | *66.9% (58.7, 74.2)* |
| HIV Pos (n=5) | 4/5 (80%; 37.5, 96.4) | *67/110 (60.9%;51.6, 69.5)* | 8.5% | 98.5% | 2.0 (1.7-2.4) | 61.7% (52.6, 70.1) |
| HIV Neg (n=24) | 21/24 (87.5%; 69.0, 95.7) |  | 32.8% | 95.7% | 2.3 (2.1-2.4) | 65.7% (57.3, 73.2) |
| Mx pos (n=8) | 6/8 (75%; 40.9, 92.8) |  | 12.2% | 97.1% | 1.9 (1.6-2.2) | 61.9% (52.9, 70.1) |
| Mx Neg/ND (n=21) | 19/21 (90.5%; 71.1, 97.3) |  | 30.6% | 97.1% | 2.3 (2.2-2.5) | 65.6% (57.2, 73.2) |
| BCG vacc. (n=10) | 6/10 (60%; 31.3, 83.2) |  | 12.2% | 94.4% | 1.5 (1.2-2.0) | 60.8% (51.9, 69.1) |
| BCG uk/no vacc. (n=19) | 19/19 (100%; 83.2, 100) |  | 30.6% | 100% | 2.6 (2.5-2.7) | 66.7% (58.1, 74.2) |
| S (+), C (+), n=4 | 4/4 (100%; 51.0, 100) |  | 8.5% | 100% | 2.6 (2.5-2.7) | 62.3% (53.1, 70.6) |
| S (-), C (+), n=25 | 21/25 (84%; 65.3, 93.6) |  | 32.8% | 94.4% | 2.2 (2.0-2.3) | 65.2% (56.83, 72.7) |
| Pos: Positive, Neg: Negative, CI: Confidence interval, PPV: Positive predictive value, NPV: Negative predictive value, LRP: likelihood ratio for positive test, DA: Diagnostic accuracy, Uk: unknown, Mx: Mantoux test, ND: not done, Vacc. -Vaccination. C: MGIT 960 culture, S: ZN stained smear, *MDR-TB cases are DST confirmed 46 from PTB and 4 from EPTB group, P value <0.001 (control vs patient), P_Rv2970c_ =<0.001, P_Rv2145c_ =0.008 and P_Rv1827_=<0.001 (sensitive vs MDR-TB). | | | | | | |

**Supplementary Table 6: Comparison of ELISA sensitivity and specificity of *M. tuberculosis*** antigens in bacteriologically confirmed MDR-TB cases (n=50; 46 PTB and 4 EPTB)

| **Antigens/group** | **Sensitivity [n (%; 95% CI)]** | | **Specificity [n (%; 95% CI)]** | **PPV (%)** | **NPV (%)** | **LRP (95% CI)** | **DA %, (95%CI)** |
| --- | --- | --- | --- | --- | --- | --- | --- |
| **Rv2970c (lipN)** | | | | | | | |
| *MDR-TB cases (n=50)* | *50/50 (100%; 92.9, 100)* | | *108/110 (98.2%;93.6,99.5)* | *96.1%* | *100%* | *55.0 (20.6-146.5)* | *98.7% (95.6, 99.6)* |
| HIV Neg (n=50) | 50/50 (100%; 92.9, 100) | | 108/110 (98.2%;93.6,99.5) | 96.1% | 100% | 55.0 (20.6-146.5) | 98.7% (95.6, 99.6) |
| Mx pos (n=34) | 34/34 (100%; 89.8, 100) | |  | 94.4% | 100% | 55.0 (20.6-146.5) | 98.6% (95.1, 99.6) |
| Mx Neg/ND (n=16) | 16/16 (100% (80.6, 100) | |  | 88.9% | 100% | 55.0 (20.6-146.5) | 98.4% (94.4, 99.6) |
| BCG vacc. (n=20) | 20/20 (100%; 83.9, 100) | |  | 90.9% | 100% | 55.0 (20.6-146.5) | 98.5% (94.6, 99.6) |
| BCG uk/no vacc. (n=30) | 30/30 (100%; 88.6, 100) | |  | 93.7% | 100% | 55.0 (20.6-146.5) | 98.6% (94.9, 99.6) |
| S (1+), C (+), n=50 | 50/50 (100%; 92.8, 100) | |  | 96.1% | 100% | 55.0 (20.6-146.5) | 98.7% (95.6, 99.7) |
| **Rv2145c (wag31)** | | | | | | | |
| *MDR-TB cases (n=50)* | *50/50 (100%; 92.9, 100)* | | *107/110 (97.3%;92.3,99.1)* | *94.3%* | *100%* | *37.7 (19.1-70.5)* | *98.1% (94.6, 99.4)* |
| HIV Neg (n=50) | 50/50 (100%; 92.9, 100) | | 107/110 (97.3%;92.3,99.1) | 94.3% | 100% | 36.7 (19.1-70.5) | 98.1% (94.6, 99.4) |
| Mx pos (n=34) | 34/34 (100%; 89.8, 100) | |  | 92.0% | 100% | 36.7 (19.1-70.5) | 97.9% (94.0, 99.3) |
| Mx Neg/ND (n=16) | 16/16 (100%; 80.6, 100) | |  | 84.2% | 100% | 36.7 (19.1-70.5) | 97.6% (93.2, 99.2) |
| BCG vacc. (n=20) | 20/20 (100% 83.9, 100) | |  | 87.0% | 100% | 36.7 (19.1-70.5) | 98.0% (93.4, 99.2) |
| BCG uk/no vacc.(n=30) | 30/30 (100%; 88.6, 100) | |  | 90.9% | 100% | 36.7 (19.1-70.5) | 97.9% (93.9, 99.3) |
| S (1+), C (+), n=50 | 50/50 (100%; 92.9, 100) | |  | 94.3% | 100% | 36.7 (19.1-70.5) | 98.1% (94.6, 99.4) |
| **Rv1827 (garA)** | | | | | | | |
| *MDR-TB cases (n=50)* | *50/50 (100%; 92.9, 100)* | | *104/110(94.5%;88.6, 97.5)* | *89.3%* | *100%* | *18.3 (13.2-25.4)* | *96.2% (92.1, 98.3)* |
| HIV Neg (n=50) | 50/50 (100%; 92.9, 100) | | 104/110(94.5%;88.6, 97.5) | 89.3% | 100% | 18.3 (13.2-25.4) | 96.2% (92.1, 98.3) |
| Mx pos (n=34) | 34/34 (100%; 89.8, 100) | |  | 85.0% | 100% | 18.3 (13.2-25.4) | 95.8% (91.2, 98.1) |
| Mx Neg/ND (n=16) | 16/16 (100%; 80.64, 100) | |  | 72.7% | 100% | 18.3 (13.2-25.4) | 95.2% (90.0, 97.8) |
| BCG vacc. (n=20) | 20/20 (100%; 83.9, 100) | |  | 76.9% | 100% | 18.3 (13.2-25.4) | 95.4% (90.3, 97.9) |
| BCG uk/no vacc. (n=30) | 30/30 (100%; 88.6, 100) | |  | 83.3% | 100% | 18.3 (13.2-25.4) | 95.7% (91.0, 98.0) |
| S (1+), C (+), n=50 | 50/50 (100%; 92.9, 100) | |  | 89.3% | 100% | 18.3 (13.2-25.4) | 96.2% (92.1, 98.3) |
| **Rv0164 (TB 18.5)** | | | | | | | |
| *MDR-TB (n=50)* | *38/50 (76.0%; 62.6, 85.7)* | | *96/110 (87.3%;79.8,92.3)* | *73.1%* | *88.9%* | *6.0 (5.1 -7.0)* | *83.7% (77.2, 88.7)* |
| HIV Neg (n=50) | 38/50 (76.0%; 62.6, 85.7) | | 96/110 (87.3%;79.8,92.3) | 73.1% | 88.9% | 6.0 (5.1-7.0) | 83.7% (77.2, 88.7) |
| Mx pos (n=34) | 22/34 ( 64.7%; 47.9, 78.5) | |  | 61.1% | 89.0% | 5.1 (4.2-6.1) | 81.9% (74.9, 87.4) |
| Mx Neg/ND (n=16) | 16/16 (100%; 80.6, 100) | |  | 53.4% | 100% | 7.9 (6.8-9.0) | 88.9% (82.2, 93.2) |
| BCG vacc. (n=20) | 11/20 (55.0%; 34.2, 74.2) | |  | 44.0% | 91.4% | 4.3 (3.2-5.7) | 82.3% (74.8, 87.9) |
| BCG uk/non vacc.(n=30) | 27/30 (90.0%; 74.4, 96.5) | |  | 65.8% | 97.0% | 7.1 (6.1-8.2) | 87.9% (81.4, 92.3) |
| S (1+), C (+), n=50 | 42/50 (84.0%; 71.5, 91.7) | |  | 75.0% | 92.3% | 6.6 (5.7-7.7) | 86.2% (80.1, 90.7) |
| **Rv1437 (pgk)** | | | | | | | |
| *MDR-TB (n=50)* | *41/50 (82.0%; 69.2, 90.2)* | | *95/110 (86.7%;78.7, 91.6)* | *73.2%* | *91.3%* | *6.0 (5.2-6.9)* | *85.0% (78.6, 89.7)* |
| HIV Neg (n=50) | 41/50 (82.0%; 69.2, 90.2) | | 95/110 (86.7%;78.7, 91.6) | 73.2% | 91.3% | 6.0 (5.2-6.9) | 85.0% (78.6, 89.7) |
| Mx pos (n=34) | 25/34 (73.5%; 56.9, 85.4) | |  | 62.5% | 91.3% | 5.4 (4.6-6.3) | 83.3% (76.4, 88.5) |
| Mx Neg/ND (n=16) | 16/16 (100%; 80.6, 100) | |  | 51.6% | 100% | 7.4 (6.4-8.4) | 88.1% (81.3, 92.6) |
| BCG vacc. (n=20) | 16/20 (80.0%; 58.4, 91.9) | |  | 51.6% | 96.0% | 5.9 (5.0-6.9) | 85.4% (78.3, 90.4) |
| BCG uk/no vacc. (n=30) | 25/30 (83.3%; 66.4, 92.7) | |  | 62.5% | 95.0% | 6.1 (5.3-7.1) | 85.7% (79.0, 90.6) |
| S (1+), C (+), n=50 | 39/50 (78.0%; 64.8, 87.2) | |  | 72.2% | 89.6% | 5.7 (5.0-6.6) | 83.7% (77.2, 88.7) |
| **Esat6 Ag** | | | | | | | |
| *MDR-TB (n=50)* | *33/50 (66.0%; 52.1, 77.6)* | | *78/110 (70.9%;61.8, 78.6)* | *50.8%* | *82.1%* | *2.3 (2.1-2.5)* | *69.4% (61.8, 76.0)* |
| HIV Neg (n=50) | 45/50 (90.0%; 78.6, 95.6) | | 78/110 (70.9%;61.8, 78.6) | 58.4% | 94.0% | 3.1 (3.0-3.3) | 76.9% (69.8, 82.7) |
| Mx pos (n=34) | 31/34 (91.2%; 77.0, 96.9) | |  | 49.2% | 96.3% | 3.1 (2.9-3.3) | 75.7% (68.1, 82.0) |
| Mx Neg/ND (n=16) | 14/16 (87.5%; 64.0, 96.5) | |  | 30.4% | 97.5% | 3.0 (2.8-3.3) | 73.0% (64.7, 80.0) |
| BCG vacc. (n=20) | 16/20 (80.0%; 58.4, 91.9) | |  | 33.3% | 95.1% | 2.7 (2.5-3.0) | 72.3% (64.1, 79.3) |
| BCG uk/no vacc. (n=30) | 29/30 (96.7%; 83.3, 99.4) | |  | 47.5% | 98.7% | 3.3 (3.1-3.5) | 76.4% (68.7, 82.7) |
| S (1+), C (+), n=50 | 45/50 (90%; (8.6, 95.6) | |  | 58.4% | 94.0% | 3.1 (2.9-3.3) | 76.9% (69.8, 82.7) |
| **38kDa Ag** | | | | | | | |
| *MDR-TB (n=50)* | *47/50 (94.0%; 83.8,97.9)* | | *67/110 (60.9%;51.6, 69.5)* | 52.2% | 95.7% | 2.5 (2.3-2.5) | 71.2% (63.8, 77.7) |
| HIV Neg (n=50) | 47/50 (94%; 83.8, 97.9) | | 67/110 (60.9%;51.6, 69.5) | 52.2% | 95.7% | 2.5 (2.3-2.5) | 71.2% (63.8, 77.7) |
| Mx pos (n=34) | 31/34 (91.2%; 77.0, 96.9) | |  | 41.9% | 95.7% | 2.3 (2.2-2.5) | 68.1% (60.1, 75.1) |
| Mx Neg/ND (n=16) | 16/16 (100%; 80.6, 100) | |  | 27.1% | 100% | 2.6 (2.4-2.7) | 65.9% (57.2, 73.6) |
| BCG vacc. (n=20) | 18/20 (90%; 69.9, 97.2) | |  | 29.5% | 97.1% | 2.3 (2.2-2.4) | 65.4% (56.9, 73.0) |
| BCG uk/no vacc. (n=30) | 29/30 (96.7%; 83.3, 99.4) | |  | 40.3% | 98.5% | 2.5 (2.4-2.6) | 68.6% (60.5, 75.7) |
| S (1+), C (+), n=50 | 47/50 (94%; 83.8, 97.9) | |  | 52.2% | 95.7% | 2.5 (2.3-2.5) | 71.2% (63.8, 77.7) |
| Pos: Positive, Neg: Negative, CI: Confidence interval, PPV: Positive predictive value, NPV: Negative predictive value, LRP: likelihood ratio for positive test, DA: Diagnostic accuracy, uk: unknown, Mx: Mantoux test, ND: not done, Vacc. -Vaccination. C: MGIT 960 culture, S: ZN stained smear, *MDR-TB cases are DST confirmed 46 from PTB and 4 from EPTB group, P value <0.001 (control vs patient), P_Rv2970c_ =<0.001, P_Rv2145c_ =0.008 and P_Rv1827_=<0.001 (sensitive vs MDR-TB). | | | | | | | |
|  | |  | | | | | |
